# Supplementary material for: Siglec-6 mediates the uptake of extracellular vesicles through a noncanonical glycolipid binding pocket
Source: Nat Commun. 2023 Apr 22;14:2327. doi: 10.1038/s41467-023-38030-6 (PMC10122656; doi:10.1038/s41467-023-38030-6)
Supplement: Supplementary file 1 — Supplementary Information [file 41467_2023_38030_MOESM1_ESM.pdf]

Supplementary Information

Siglec-6 mediates the uptake of extracellular vesicles through a noncanonical glycolipid binding pocket

Edward N. Schmidt[1], Dimitra Lamprinaki[1], Kelli A. McCord[1], Maju Joe[1], Mirat Sojitra[1], Ayk Waldow[1], Jasmine Nguyen[2], John Monyror [3,4], Elena N. Kitova[1], Fahima Mozaneh[1], Xue Yan Guo[1], Jaesoo Jung[1], Jhon R. Enterina[5], Gour C. Daskhan[1], Ling Han[1], Amanda R. Krysler[3], Christopher R. Chromwell[3], Basil P. Hubbard[3#], Lori J. West[5,6,7], Marianne Kulka[5,8], Simonetta Sipione[3,4], John S. Klassen[1], Ratmir Derda[1], Todd L. Lowary[1,9,10], Lara K. Mahal[1], Meghan R. Riddell[2], Matthew S. Macauley[1,5,*]

[1]Department of Chemistry, University of Alberta, Edmonton, Alberta, Canada

[2]Department of Obstetrics & Gynaecology and Physiology University of Alberta, Edmonton, Alberta, Canada

[3]Department of Pharmacology, University of Alberta, Edmonton, Alberta, Canada

[4]Neuroscience and Mental Health Institute, University of Alberta, Edmonton, Alberta, Canada

[5]Department of Medical Microbiology and Immunology, University of Alberta, Edmonton, Alberta, Canada

[6]Department of Pediatrics, University of Alberta, Edmonton, AB, Canada

[7]Department of Surgery, University of Alberta, Edmonton, AB, Canada

[8]National Research Council, Edmonton, Alberta, Canada

[9]Institute of Biological Chemistry, Academia Sinica, Nangang, Taipei, Taiwan

[10]Institute of Biochemical Sciences, National Taiwan University, Taipei, Taiwan

*Corresponding Address: 11227 Saskatchewan Dr., University of Alberta, macauley@ualberta.ca

[#]Present address: Department of Pharmacology and Toxicology, University of Toronto, Toronto, ON, Canada

Synthetic Schemes and Chemical Characterization

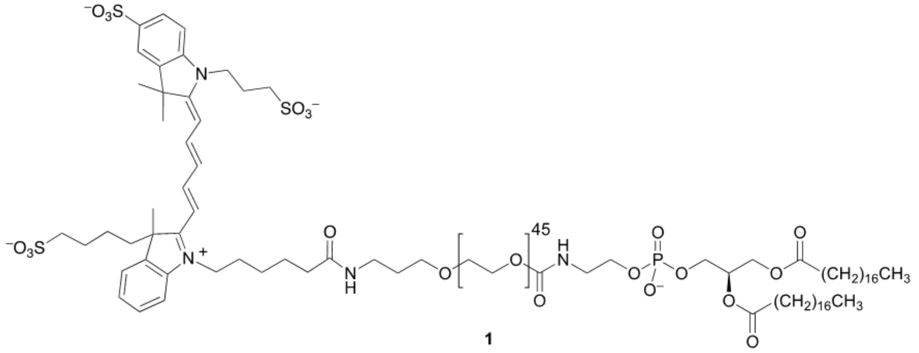

1

AF647–PEG$_{45}$–DSPE 1 was synthesized as described by Bhattacherjee *et al.*.[1]

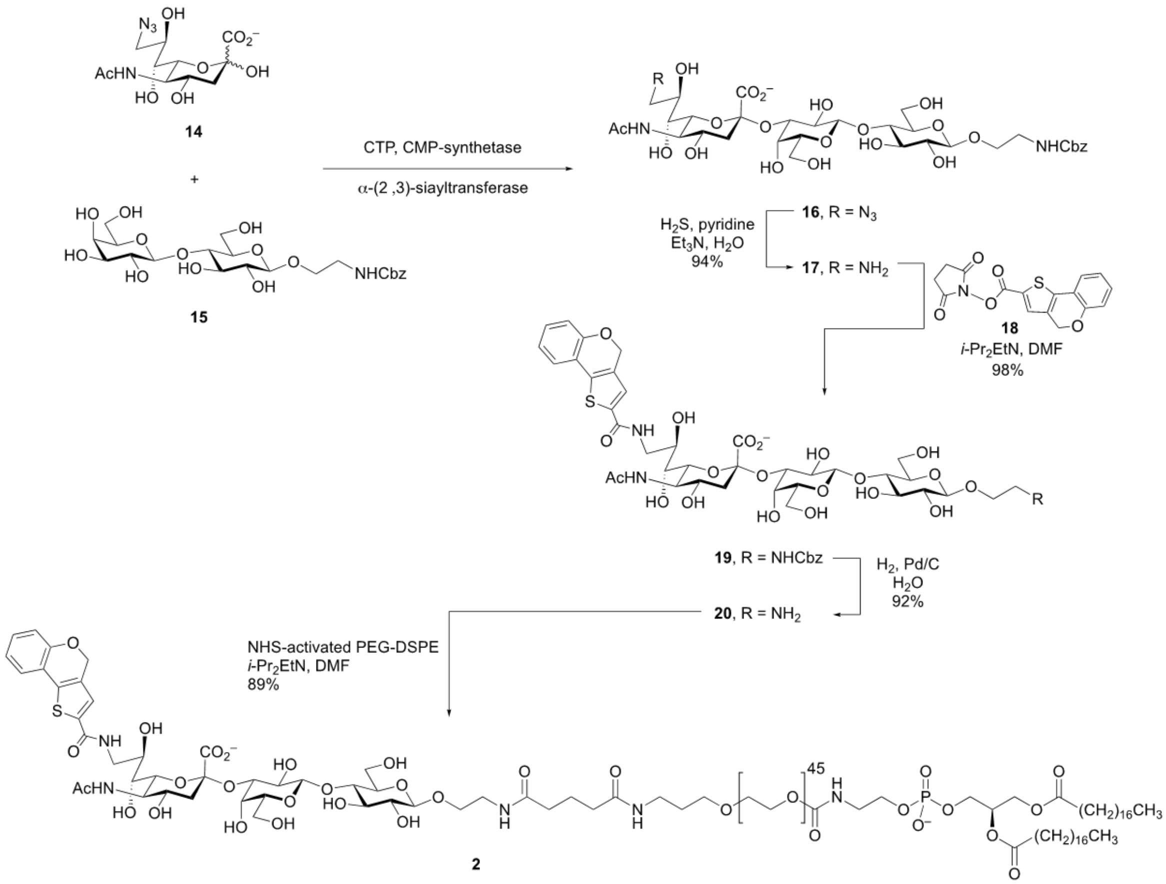

Siglec-1 ligand–PEG_{45}–DSPE 2:

A mixture of 9-azido sialic acid derivative **14**[2] (10 mg, 0.029 mmol), CTP (31 mg, 0.062 mmol), 12 mM M $MgCl_2$ in Tris-HCl buffer (pH 8.6, 285 mL) was treated with CMP-synthetase (25 μL) and the pH of the solution was adjusted to ~9.0 to 9.5 by adding 75 mL of aqueous 1M NaOH and the solution was incubated at 37 °C for 30 min. The progress of the reaction was monitored by TLC and when complete disaccharide **15**[3] (19.3 mg, 0.037 mmol), 0.5 M $MgSO_4$ and of Tris-HCl buffer (pH 8.6, 560 mL, final concentration 15 mM) and α-(2 →3)-sialyltransferases (0.15 mg/mL, PmSTI) were added. The reaction mixture was further incubated at 37 °C for 2 h. The excess enzymes were quenched by the addition of EtOH (500 μL), the mixture was centrifuged, crude product was washed with EtOH (2 x 100 mL) and the supernatants were collected. The EtOH in the supernatant was removed under reduced pressure and the resulting aqueous solution was frozen at –80 °C and lyophilized to afford a white solid. The crude residue was subjected to P2-gel filtration chromatography using H_2O as eluent to afford **16** (22.8 mg, 90 %) as a white powder after lyophilization; ^1H NMR (600 MHz, D_2O) δ (ppm) 7.41–7.37 (m, 5H), 5.09 (s, 2H), 4.72 (d, 1H, *J* = 8.0 Hz), 4.42 (d, 1H, *J* = 8.0 Hz), 4.04 (ddd, 1H, *J* = 21.0, 12.0, 3.0 Hz), 3.94–3.90 (m, 4H), 3.81–3.76 (m, 3H), 3.70

(t, 1H, J = 10.0 Hz), 3.65–3.40 (m, 12H), 3.39 (dd, 1H, J = 15.0, 7.8 Hz), 2.98 (dd, 1H, J = 13.2, 9.6 Hz), 2.67 (dd, 1H, J = 12.6, 4.8 Hz), 1.99 (s, 3H, NHCOC<u>H</u>$_3$), 1.70 (t, J = 12.6 Hz, 1H). HRMS (ESI) calculated for *m/z* $[M - H]^+$ cald for $C_{33}H_{49}N_5O_{20}$: 835.2971, found: 834.2889.

Trisaccharide **16** (10 mg, 0.011 mmol) was dissolved in a mixture of pyridine (1.8 mL), Et_3N (125 μL) and H_2O (125 μL) and the reaction mixture were cooled to 0 °C in an ice bath for approximately 15 min. H_2S gas was bubbled through the solution for 5 min, the reaction mixture turned an intense blue colour and the round bottom flask was capped and stirred overnight at room temperature. After completion of the reaction, the solvent was evaporated, the crude product was dissolved in methanol (10 mL) and the resulting solution was centrifuged. The supernatant was collected, concentrated and the crude mixture was purified by P2-gel filtration chromatography using H_2O as eluent to afford **17** (9.1 mg, 94 %) as a white powder after lyophilization. HRMS (ESI) calculated for *m/z* $[M - H]^+$ cald for $C_{33}H_{51}N_3O_{20}$: 808.2993, found: 808.3005.

Amine **17** (5.0 mg, 6.18 mmol, 1 equiv.) and NHS-activated ester **18**[4] (2.5 mg, 7.72 μmol, 1.25 equiv.) were dissolved in anhydrous DMF (0.5 mL). *N*,*N*-Diisopropylethylamine (1.87 mL, 18.54 mmol, 3 equiv.) was added to the reaction mixture, and the solution was stirred overnight at room temperature. DMF was removed under reduced pressure and the residue was co-evaporated with toluene (2 x 5 mL). The crude product was dissolved in H_2O (5 mL) and hydrolyzed **18** was separated from the desired product **19** through successive extraction with EtOAc (5 mL, 5–6 times). TLC confirmed removal of hydrolyzed **18**. The aqueous layers were collected and lyophilized to afford **19** (5.52 mg, 98 %) as a white powder; ^1H NMR (500 MHz, D_2O**)** δ (ppm) 7.82 (t, 1H, J = 7.5 Hz), 7.76–7.72 (m, 2H), 7.68–7.66 (m, 2H), 7.42–7.40 (m, 5H), 5.10 (s, 2H), 4.49 (J = 8.0 Hz, 1H), 4.43 (d, 1H, J = 8.0 Hz,), 4.08 (dd, 1H, J = 10.0, 3.5 Hz,), 4.06–4.04 (m, 2H), 3.94–3.85 (m, 4H), 3.78–3.52 (m, 15H), 3.39–3.35 (m, 2H), 3.01 (dd, 1H, J = 12.5, 9.5 Hz), 2.73 (dd, 1H, J = 12.5, 4.5 Hz), 2.01 (s, 3H, NHCOC<u>H</u>$_3$), 1.78 (t, 1H, J = 12.5 Hz). HRMS (ESI) calculated for *m/z* $[M - H]^+$ cald for $C_{45}H_{56}N_3O_{22}S$: 1023.3154, found: 1023.3145.

Trisaccharide **19** (5.0 mg, 4.88 mmol) was dissolved in H_2O (2 mL), followed by addition of Pd/C (10 mg, 5 mol%). Hydrogen gas was bubbled through the solution and the mixture was stirred overnight under a hydrogen atmosphere. After completion of reaction, the catalyst was removed by filtration though

a Celite pad. The solvent was evaporated, and the mixture was purified using a C18 column (gradient elution from H_2O to MeOH/H_2O (10 % to 50 %, V/V)) to produce **20** (4.0 mg, 92 %) as while solid after lyophilization of the fractions containing the desired product; ^{1}H NMR (600 MHz, D_2O) δ (ppm) 7.38–7.37 (m, 2H), 7.24 (t, 1H, *J* = 7.4 Hz), 7.01 (t, 1H, *J* = 7.4 Hz), 6.93 (d, 1H *J* = 7.8 Hz), 5.19 (s, 2H), 4.34 (d, 2H, *J* = 7.8 Hz), 4.03 (dd, 1H, *J* = 9.6, 3.0 Hz), 3.96 (ddd, 1H, *J* = 11.4, 9.6, 3.0 Hz), 3.88 (d, 1H, *J* = 3.0 Hz), 3.83–3.80 (m, 4H), 3.70–3.64 (m, 8H), 3.56–3.46 (m, 8H), 3.44–3.32 (m, 2H), 3.16 (dd, 1H, *J* = 12.5, 9.5 Hz), 2.01 (t, 1H, *J* = 4.8 Hz), 2.73 (dd, 1H, *J* = 12.5, 4.8 Hz), 1.99 (s, 3H, NHCOC$\underline{H}_3$), 1.75 (t, 1H, *J* = 12.5 Hz). ^{13}C NMR (125 MHz, D_2O) δ (ppm) 176.0, 174.6, 164.8, 152.8, 137.8, 137.1, 133.3, 131.4, 127.5, 124.8, 123.8, 120.2, 117.6, 103.7, 102.88, 100.9, 79.1, 77.0, 76.3, 75.6, 75.0, 73.9, 73.6, 71.9, 70.9, 70.2, 69.2, 68.3, 67.6, 66.7, 63.5, 62.0, 60.8, 52.7, 49.9, 43.8, 40.5, 23.0. HRMS (ESI-MS) calculated for *m/z* [M + Na]$^+$ cald for $C_{37}H_{51}N_3NaO_{20}S$: 912.2679, found: 912.2681.

A mixture of **20** (1 mg, 0.91 mmol, 1.25 equiv.) and NHS-activated PEG-DSPE (2.5 mg, 0.83 mmol, 1 equiv.) were dissolved in anhydrous DMF (100–150 µL, ~10 mM) and placed in a 0.5 mL centrifuge tube at room temperature. The reaction mixture was degassed with N_2. A diluted solution of DIPEA (1.50–2.0 equiv.) in dry DMF was added carefully to adjust pH of the solution ~8.0 and the reaction mixture was stored at room temperature overnight. An aliquot of the reaction mixture was taken for TLC ($CHCl_3$–MeOH–H_2O = 75:23:2) analysis. The coupling was performed under anhydrous condition to avoid hydrolysis of the NHS-activated PEG-DSPE. The solvent was removed under *vacuum*, and the crude product was dissolved in water. The crude product was loaded to Sephadex G-100 gel filtration column using H_2O as an eluent to afford **20**-PEG-DSPE conjugate as a white powder after lyophilization of fractions having the desired product. Yield: (3.8 mg, 89 %), coupling efficiency 62 %.

^{1}H NMR (600 MHz, $MeOD_4$): δ 7.49 (d, *J* = 7.8 Hz, 1H), 7.38-7.33 (m, 1H), 7.19 (t, *J* = 7.8 Hz, 1H), 6.97 (t, *J* = 7.8 Hz, 1H), 7.99 (d, *J* = 7.8 Hz, 1H), 5.25 (s, 2H), 4.44 (d, *J* = 8.0 Hz, 1H), 4.42 (d, *J* = 8.0 Hz, 1H), 4.31 (d, *J* = 7.8 Hz, 1H), 4.19-3.98 (m, 2H), 4.97 (t, *J* = 5.4 Hz, 1H), 3.90-4.86 (m, 2H), 3.74-3.71 (m, 2H), 3.64 (broad s, 123H), 3.3-3.34-3.22 (m, 8H), 2.35-2.29 (m, 4H), 2.23-2.19 (m, 4H), 1.99 (s, 3H), 1.89-1.86 (m, 2H), 1.76-1.73 (m, 2H), 1.60-1.58 (m, 2H), 1.28 (broad s, 64H), 0.89 (s, 6H); The MALDI-TOF-MS spectrum showed the average mass centered at 3.8 kDa and the expected average mass was 3.8 kDa. The coupling efficiency was determined through assigning underline signals of the aromatic protons signals

at 7.49 ppm (d), 7.38–7.33 ppm (m), 6.97 (t) and 7.99 (d) of the phenyl moieties at C9 position of bifunctionally substituted Neu5Ac with terminal methyl groups at 0.89 ppm (s) of the DSPE lipid.

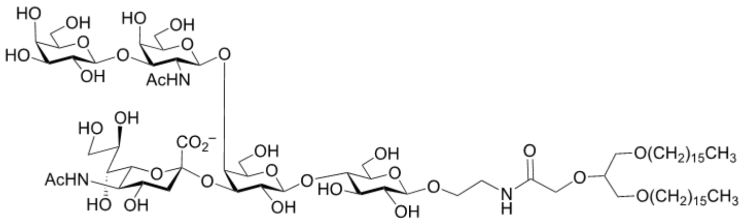

3

Neoglycolipid 3 was prepared as described previously by Han et *al*.[5]

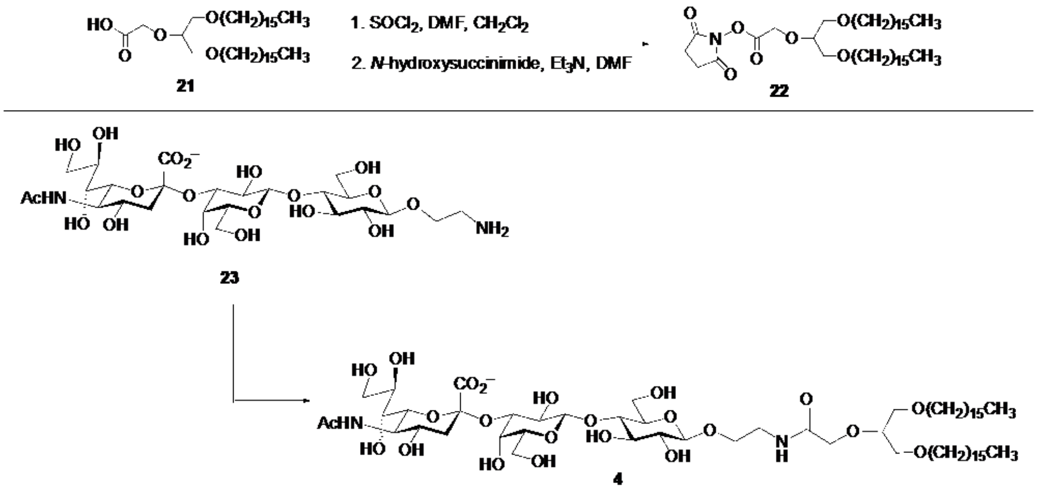

Neoglycolipid 4. To a stirred solution of acid **21** (8.0 mg, 13.4 μmol) in CH_2Cl_2 (3 mL) were added thionyl chloride (5.0 μL, 68.0 μmol) and dry DMF (20.0 μL) successively at room temperature. After heating at reflux overnight, the solution was cooled, the solvent was evaporated, and the residue was dried under high vacuum (2.5 h). The dried acyl chloride was dissolved in CH_2Cl_2 (3 mL), *N*-hydroxysuccinimide (6.0 mg, 52.0 μmol) and triethylamine (5.0 μL, 36.0 μmol) were added at 0 °C and the solution was heated at reflux overnight. After cooling to room temperature, the solution was diluted with CH_2Cl_2 (5 mL), washed with brine, dried over Na_2SO_4, filtered, and the filtrate was concentrated to give the NHS-ester **22**, which was used directly for the next step without any further purification.

Trisaccharide amine **23**[6] (2.0 mg, 3.0 μmol) was dissolved in *N,N*-dimethylacetamide (0.4 mL), and *N*,*N*-diisopropylethylamine (5 μL, 28.0 μmol) was added. This solution was then added to a glass vial containing **22** followed by THF (0.3 mL) and the mixture was stirred overnight at room temperature. The reaction mixture was directly loaded and purified by size exclusion column chromatography (Sephadex-LH20, CH_2Cl_2–CH_3OH, 1:1) to afford **4** (0.9 mg, 25 %). R_f 0.3, EtOAc–CH_3OH–HOAc–H_2O (36:9:9:6); ^1H NMR (700 MHz, CD_3OD plus a few drops of $CDCl_3$, δ_H) 4.42 (d, 1H, *J* = 7.8 Hz, H-1), 4.31 (d, 1H, *J* = 7.8 Hz, H-1), 4.15–4.10 (m, 2H), 4.05 (dd, 1H, *J* = 3.1, 9.7 Hz), 3.96–3.90 (m, 3H), 3.90–3.82 (m, 3H), 3.80-3.74 (m, 2H), 3.74–3.40 (m, 24H), 3.26 (dd, 1H, *J* = 7.9, 7.9 Hz), 3.21 (ddd, 2H, *J* = 1.6, 3.2, 4.8 Hz), 2.85 (dd, 1H, *J* = 4.2, 12.5 Hz), 2.79 (dd, 1H, *J* = 7.5, 7.5 Hz), 2.56 (dd, 1H, *J* = 7.5, 7.5 Hz), 2.00 (s, 3H, NHCOC$\underline{H}_3$), 1.75–1.60 (m, 5H), 1.40–1.20 (m, 56H), 0.86 (dd, 6H, *J* = 6.9 Hz); HRMS (ESI) calcd. for $[M - H]^-$ $C_{62}H_{116}N_2O_{23}$ 1255.7896, found 1255.7909.

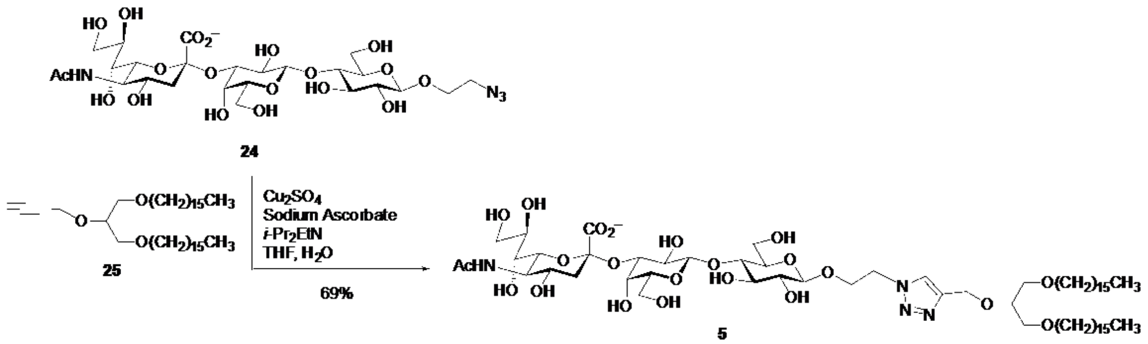

Neoglycolipid 5. To a stirred solution of trisaccharide azide **24**[7] (8.0 mg, 11.0 µmol) and alkyne **25**[67] (15.0 mg, 26.0 µmol) in a mixture of THF (3 mL) and water (3 mL) at room temperature were added *N*,*N*-diisopropylethylamine (6.0 µL, 34.0 µmol), copper (II) sulfate pentahydrate (53.0 mg, 212.0 µmol) and L-ascorbic acid sodium salt (81.0 mg, 408.0 µmol) successively. The reaction mixture was shielded from light (aluminum foil) and stirred overnight. The reaction mixture was then concentrated, and the residue was purified by size exclusion column chromatography (Sephadex-LH-20, CH_2Cl_2–CH_3OH, 1:1) to afford **5** (10.0 mg, 69 %). R_f 0.3, EtOAc–CH_3OH–HOAc–H_2O (36:9:9:6); ^{1}H NMR (700 MHz, CD_3OD plus a few drops of $CDCl_3$, δ_H) 8.03 (s, 1H), 4.65–4.55 (m, 2H), 4.39 (d, 1H, *J* = 7.0 Hz, H-1), 4.31 (d, 1H, *J* = 7.8 Hz, H-1), 4.24–4.20 (m, 2H), 3.95–3.40 (m, 28H), 3.22–3.20 (m, 1H), 2.90–2.80 (m, 1H), 2.10–2.00 (m, 2H), 1.99 (s, 3H, NHCOC$\underline{H}_3$), 1.70–1.50 (m, 5H), 1.40–1.20 (m, 56H), 0.86 (dd, 6H, *J* = 6.9 Hz); HRMS (ESI) calcd. for $[M - H]^-$ $C_{63}H_{116}N_4O_{22}$ 1279.8008, found 1279.8014.

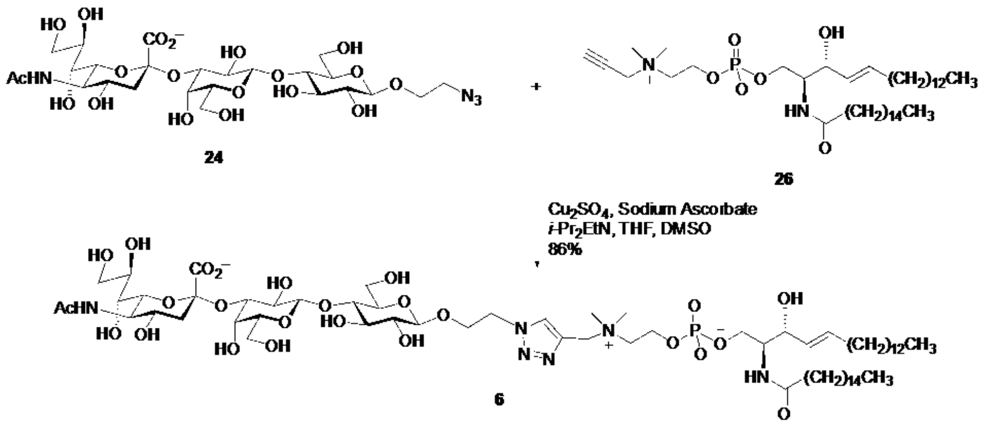

Neoglycolipid 6. To a stirred solution of trisaccharide azide **24**[7] (2.2 mg, 3.1 μmol) in water (0.5 mL), alkyne **26** (Avanti Polar Lipids, Inc., USA) (1.0 mg, 1.4 μmol) in a mixture of THF (0.3 mL) and DMSO (0.5 mL) was added at room temperature followed by *N*,*N*-diisopropylethylamine (3.0 μL, 17.0 μmol). Copper (II) sulfate pentahydrate (21.0 mg, 84.0 μmol) and L-ascorbic acid sodium salt (37.0 mg, 186.0 μmol) were then added successively. The reaction mixture was shielded from light (aluminum foil) and stirred overnight. After 24 h, copper (II) sulfate pentahydrate (14.0 mg, 56.0 μmol), and L-ascorbic acid sodium salt (30.0 mg, 151.0 μmol) each dissolved in 0.1 mL water were added again successively and stirring continued overnight. The reaction mixture was then diluted with CH_3OH–H_2O (1:1, 0.2 mL) stirred well and filtered through a cotton plug to remove most of the insoluble salts. The filtrate was directly loaded on to a C-18 column and purified by gradient elution (H_2O to CH_3OH–H_2O to neat CH_3OH) to afford **6** (1.7 mg, 86 %). R_f 0.1, EtOAc–CH_3OH–HOAc–H_2O (18:9:9:6); ^1H NMR (700 MHz, CD_3OD plus a few drops of $CDCl_3$, δ_H) 8.4 (s, 1H), 5.74–5.66 (m, 1H), 5.48–5.42 (m, 1H), 4.75 (s, 2H), 4.73–4.70 (m, 2H), 4.56 (s, 1H), 4.42 (d, 1H, *J* = 7.6 Hz, H-1), 4.39–4.34 (m, 2H), 4.32 (d, 1H, *J* = 7.8 Hz, H-1), 4.28–4.24 (m, 1H), 4.20–4.10 (m, 1H), 4.10–3.40 (m, 28H), 3.23–3.20 (m, 2H), 3.20 (s, 3H), 3.23–3.20 (m, 2H), 2.90–2.84 (m, 1H), 2.65 (s, 3H), 2.22-2.14 (m, 2H), 2.06–2.02 (m, 2H), 2.00 (s, 3H, NHCOC$\underline{H}_3$), 1.76–1.50 (m, 5H), 1.40–1.20 (m, 52H), 0.90 (dd, 6H, *J* = 6.9 Hz); HRMS (ESI) calcd. for $[M - H]^-$ $C_{66}H_{121}N_6O_{25}P$ 1427.8046, found 1427.8048.

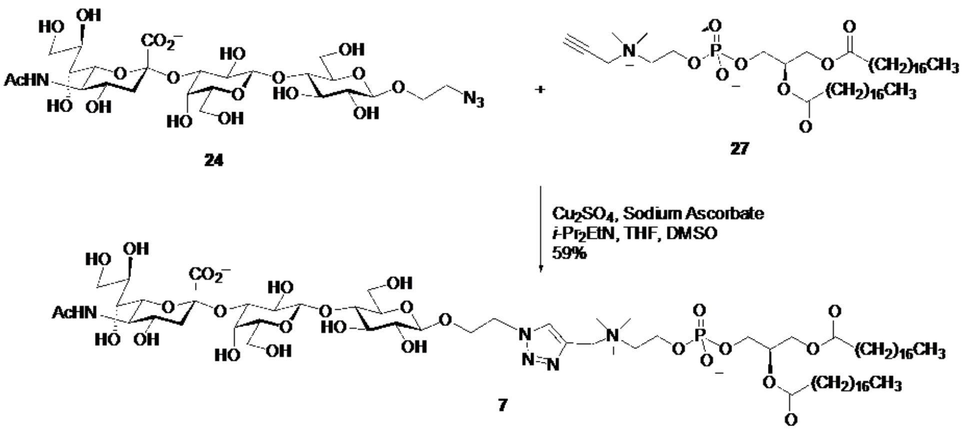

Neoglycolipid 7. To a stirred solution of trisaccharide azide **24**[7] (2.2 mg, 3.1 μmol) in water (0.6 mL), alkyne **27** (Avanti Polar Lipids, Inc., USA) (1.0 mg, 1.2 μmol) in a mixture of THF (0.3 mL) and DMSO (0.5 mL) was added at room temperature followed by *N*,*N*-diisopropylethylamine (3.0 μL, 17.0 μmol). Copper (II) sulfate pentahydrate (23.0 mg, 92.0 μmol) and L-ascorbic acid sodium salt (54.0 mg, 272.0 μmol) were then added successively. The reaction mixture was shielded from light (aluminum foil) and stirred overnight. After 24 h, copper (II) sulfate pentahydrate (16.0 mg, 64.0 μmol), and L-ascorbic acid sodium salt (34.0 mg, 171.0 μmol) each dissolved in 0.05 mL water were added again successively along with another 0.05 mL each of THF and DMSO and stirring continued overnight. The reaction mixture was then diluted with CH_3OH–H_2O (1:1, 0.2 mL) stirred well and filtered through a cotton plug to remove most of the insoluble salts. The filtrate was directly loaded onto a C-18 column and purified by gradient elution (H_2O to CH_3OH–H_2O to neat CH_3OH) to afford **7** (1.1 mg, 59 %). R_f 0.21, EtOAc–CH_3OH–HOAc–H_2O (18:9:9:6); ^{1}H NMR (700 MHz, CD_3OD plus a few drops of $CDCl_3$, δ_H) 8.5 (s, 1H), 5.38–5.40 (m, 2H), 5.40–5.32 (m, 1H), 4.76 (s, 2H), 4.73–4.70 (m, 2H), 4.48–4.43 (m, 2H), 4.42 (d, 1H, *J* = 7.9 Hz, H-1), 4.39–4.34 (m, 2H), 4.32 (d, 1H, *J* = 7.9 Hz, H-1), 4.24–4.17 (m, 1H), 4.10–3.40 (m, 28H), 3.24–3.19 (m, 2H), 3.18 (s, 3H), 2.90-2.84 (m, 1H), 2.65 (s, 3H), 2.36-2.30 (m, 2H), 2.22–2.16 (m, 2H), 2.00 (s, 3H, NHCOC$\underline{H}_3$), 1.66–1.50 (m, 6H), 1.40–1.20 (m, 60H), 0.90 (dd, 6H, *J* = 6.9 Hz); HRMS (ESI) calcd. for $[M-H]^-$ $C_{71}H_{130}N_5O_{27}P$ 1514.8618, found 1514.8631.

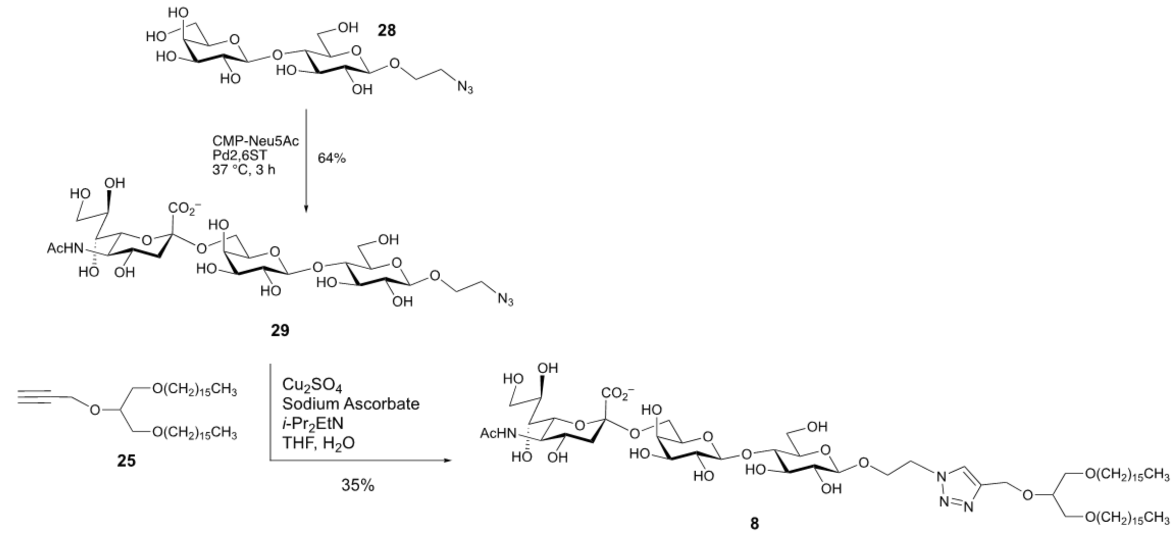

Neoglycolipid 8. Lactoside **28**[8] (1.5 mg, 0.012 mmol), CMP-Sialic acid (3.4 mg, 0.016 mmol), and $MgSO_4$ (10 mM) were dissolved in Tris-HCl buffer (100 mM, 340 µl, pH 8.8). Pd2,6ST (0.15 mg/mL) recombinant shrimp alkaline phosphatase[9] (1 µL) were added to the mixture and the reaction was placed in a shaking incubator (37 °C, 3 h). The reaction was monitored using TLC in *i*-PrOH–NH_4OH–H_2O (5:2:1) and stopped by dilution with 4 volumes of cold 95 % ethanol. The precipitated protein was centrifuged (3700 rcf, 15 min) and the supernatant was carefully decanted into a round bottom flask and concentrated. The residue was resuspended in Milli-Q water and purified on a P2 gel filtration equilibrated in Milli-Q water giving compound **29** (1.1 mg, 64 %). ^{1}H NMR (700 MHz, D_2O) δ = 4.54 (d, 1H, *J* = 7.7 Hz), 4.42 (d, 1H, *J* = 7.7 Hz), 4.02–4.08 (m, 1H), 3.94–4.01 (m, 5H), 3.78–3.93 (m, 4H), 3.59–3.77 (m, 5H), 3.49–3.58 (m, 6H), 3.42–3.62 (m, 2H), 2.74 (dd, 1H, *J* = 7.7, 12.6 Hz), 2.03 (s, 3 H), 1.80 (app t, 1H, *J* = 11.9 Hz). HRMS (ESI) calcd for *m/z* $[M - H]^-$ $C_{25}H_{42}N_4O_{19}$ 702.2443, found 701.2352.

To a stirred solution of trisaccharide azide **29** (1.1 mg, 1.6 µmol) in water (0.4 mL) was added a separately prepared solution of alkyne **25**[67] (3.1 mg, 5.3 µmol) and *N*,*N*-diisopropylethylamine (1 µL, 5.7 µmol) in THF (0.4 mL). Additional THF (0.2 mL) was added followed by successive additions of copper (II) sulfate pentahydrate (13 mg, 52.0 µmol; in 0.1 mL water) and L-ascorbic acid sodium salt (26 mg, 131.0 µmol; in 0.1 mL water). The reaction mixture was shielded from light (aluminum foil) and stirred overnight. The reaction mixture was then transferred to another flask and concentrated under vacuum and a solution of CH_2Cl_2–CH_3OH (1:1, 10 mL) was added. The sides of the flask were scraped well to ensure complete

dissolution of the product and filtered through a cotton plug. The filtrate was concentrated, and the residue was purified by size exclusion column chromatography (Sephadex-LH-20, CH_2Cl_2–CH_3OH, 1:1) to afford **8** (0.7 mg, 35 %). R_f 0.23, EtOAc–CH_3OH–HOAc–H_2O (36:9:9:6); ^1H NMR (700 MHz, CD_3OD plus a few drops of $CDCl_3$, δ_H) 8.09 (s, 1H), 4.75 (s, 2H), 4.66–4.62 (m, 2H), 4.35 (d, 1H, *J* = 7.8 Hz, H-1), 4.31 (d, 1H, *J* = 7.7 Hz, H-1), 4.25–4.17 (m, 2H), 4.05–3.35 (m, 28H), 3.18 (dd, 2H, *J* = 0, 0 Hz), 2.90–2.80 (m, 1H), 2.10–2.05 (m, 2H), 2.0 (s, 3H, NHCOC$\underline{H}_3$), 1.70–1.45 (m, 6H), 1.40-1.16 (m, 56H), 0.86 (dd, 6H, *J* = 6.9 Hz); HRMS (ESI) calcd. for $[M - H]^-$ $C_{63}H_{116}N_4O_{22}$ 1279.8008, found 1279.8018.

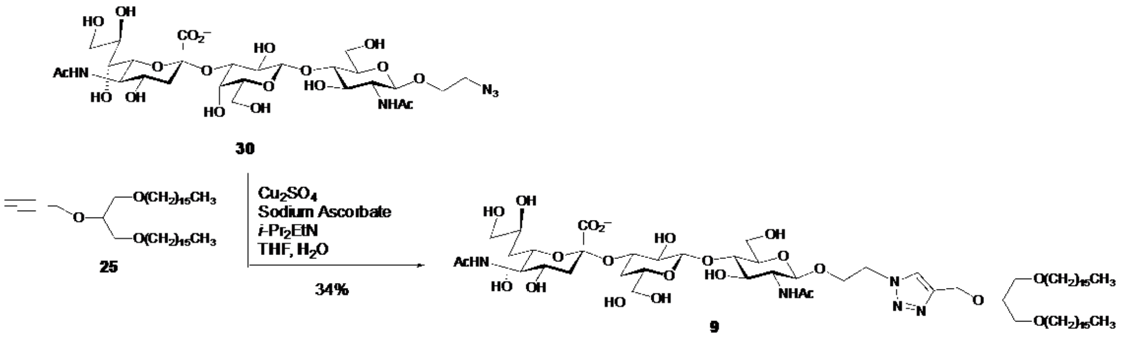

Neoglycolipid 9. This compound was prepared from trisaccharide azide **30**[10] (1.0 mg, 1.3 µmol), alkyne **25**[67] (3.2 mg, 5.5 µmol), *N*,*N*-diisopropylethylamine (1 µL, 5.7 µmol), copper (II) sulfate pentahydrate (13 mg, 52.0 µmol; in 0.1 mL water) and L-ascorbic acid sodium salt (26 mg, 131.0 µmol; in 0.1 mL water) as described for the preparation of **8** to afford **9** (0.6 mg, 34 %). R_f 0.25, EtOAc–CH_3OH–HOAc–H_2O (36:9:9:6); ^{1}H NMR (700 MHz, CD_3OD plus a few drops of $CDCl_3$, δ_H) 7.91 (s, 1H), 4.75 (s, 2H), 4.44 (d, 1H, *J* = 7.8 Hz, H-1), 4.37 (d, 1H, *J* = 8.4 Hz, H-1), 4.26–4.20 (m, 1H), 4.04 (dd, 1H, *J* = 3.0, 9.7 Hz), 3.97–3.82 (m, 4H), 3.80-3.40 (m, 24H), 3.21 (ddd, 2H, *J* = 1.7, 3.3, 5.0 Hz), 2.85 (dd, 1H, *J* = 12.2, 4.7 Hz), 2.10–2.05 (m, 2H), 2.0 (s, 3H, NHCOC$\underline{H}_3$), 1.91 (s, 3H, NHCOC$\underline{H}_3$), 1.70-1.50 (m, 6H), 1.40–1.20 (m, 56H), 0.86 (dd, 6H, *J* = 6.9 Hz); HRMS (ESI) calcd. for $[M - H]^-$ $C_{65}H_{119}N_5O_{22}$ 1320.8274, found 1320.8280.

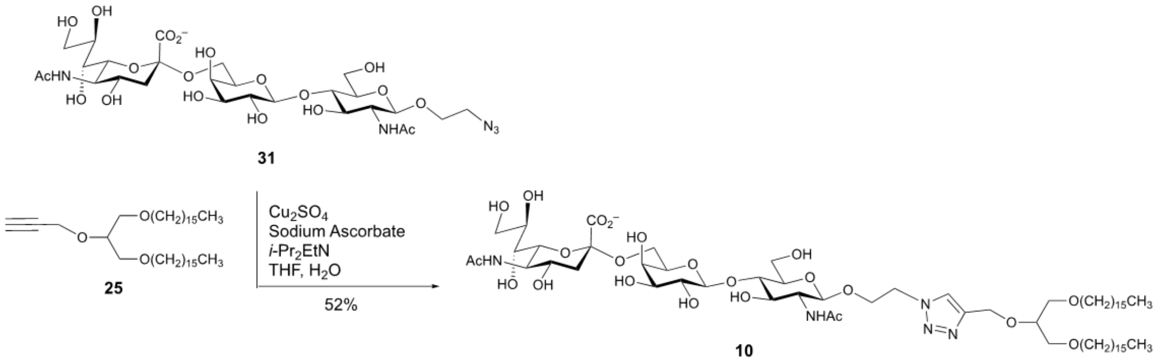

Neoglycolipid 10. This compound was prepared from trisaccharide azide **31**[10] (2.4 mg, 3.2 μmol), alkyne **25**[67] (5.8 mg, 10.0 μmol), *N*,*N*-diisopropylethylamine (3 μL, 17.0 μmol), copper (**II**) sulfate pentahydrate (21 mg, 84.0 μmol; in 0.1 mL water) and L-ascorbic acid sodium salt (37 mg, 186.0 μmol; in 0.1 mL water) as described for the preparation of **8** to afford **10** (2.2 mg, 52 %). R_f 0.18, EtOAc–CH_3OH–HOAc–H_2O (36:9:9:6); ^{1}H NMR (700 MHz, CD_3OD plus a few drops of $CDCl_3$, δ_H) 7.95 (s, 1H), 4.75 (s, 2H), 4.63–4.55 (m, 2H), 4.56 (d, 1H, *J* = 8.4 Hz, H-1), 4.32 (d, 1H, *J* = 7.7 Hz, H-1), 4.26–4.20 (m, 1H), 4.03 (dd, 1H, *J* = 9.6, 9.6 Hz), 3.94–3.78 (m, 4H), 3.80–3.40 (m, 24H), 3.21 (ddd, 2H, *J* = 1.7, 3.3, 5.0 Hz), 2.78 (dd, 1H, *J* = 4.5, 12.1 Hz), 1.99 (s, 3H, NHCOC$\underline{H}_3$), 1.94 (s, 3H, NHCOC$\underline{H}_3$), 1.70–1.50 (m, 5H), 1.40–1.20 (m, 56H), 0.89 (dd, 6H, *J* = 6.9 Hz); HRMS (ESI) calcd. for $[M - H]^-$ $C_{65}H_{119}N_5O_{22}$ 1320.8274, found 1320.8271.

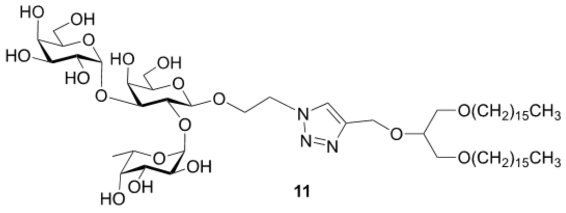

11

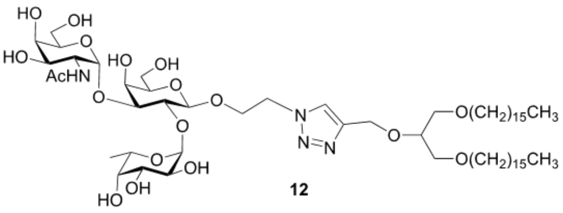

12

Neoglycolipids 11 and **12**, which were prepared as described by Han et *al.*.[5]

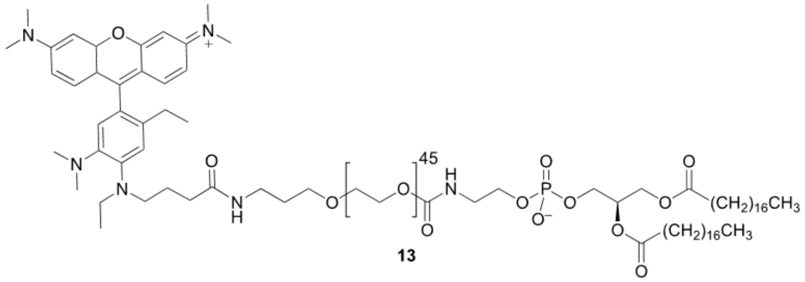

13

pHrodo-PEG$_{45}$-DSPE 13: **13** was prepared as described by Bhattacherjee et *al.*[1]

Supplementary Tables

Supplementary Table 1: Incorporation efficiency of ganglioside into liposome measure via mass spectrometry.

Ganglioside	Expected mol%	Observed mol%	Incorporation Efficiency
GM1	3.0[11]	2.88[11]	0.96[11]
GM2	3.0	2.94	0.98
GM3	3.0	2.88	0.96

Supplementary Table 2: **Mutagenesis primers for the human Siglec family.**

Primer Name	Sequence
Fwd Sig-1	AGCAGCGCTAGCATGGGCTTCTTGCCCAAGCTTC
Rvs Sig-1	AGCAGCACCGGTTCAGCCCAGGGGTGGGGCAC
Fwd Sig-1 R116A	CTCTGGTTCCTACAACTTCGCCTTCGAAATCAGTGAGGTC
Rvs Sig-1 R116A	GACCTCACTGATTTCGAAGGCGAAGTTGTAGGAACCAGAG
Fwd CD22	AGCAGCGCTAGCATGCATCTCCTCGGCCCCTGG
Rvs CD22	AGCAGCACCGGTTCAATGTTTGAGGATCACATAG
Fwd CD22 R120A	GTGGTCAGCTGGGGCTGGCGATGGAGTCCAAGACTGAG
Rvs CD22 R120A	CTCAGTCTTGGACTCCATCGCCAGCCCCAGCTGACCAC
Fwd CD33	AGCAGCGCTAGCATGCCGCTGCTGCTACTGCTG
Rvs CD33	AGCAGCACCGGTTCACTGGGTCCTGACCTCTG
Fwd CD33 R119A	GATAATGGTTCATACTTCTTTGCGATGGAGAGAGGAAGTACC
Rvs CD33 R119A	GGTACTTCCTCTCTCCATCGCAAAGAAGTATGAACCATTATC
Fwd Sig-4	AGCAGCGCTAGCATGATATTCCTCACGGCACTG
Rvs Sig-4	AGCAGCACCGGTTCAGTGAGATTCCAGGGTAG
Fwd Sig-4 R118A	GGCGGGAAGTACTACTTCGCTGGGGACCTGGGCGGCTAC
Rvs Sig-4 R118A	GTAGCCGCCCAGGTCCCCAGCGAAGTAGTACTTCCCGCC
Fwd Sig-5	AGCAGCGCTAGCATGCTGCCCCTGCTGCTGCTG
Rvs Sig-5	AGCAGCACCGGTTCACTTGCTTGTCTTGATCTC
Fwd Sig-5 R119A	CACGGGAAGCTATTTCTTCGCCGTGGAGAGAGGAAGGGATG
Rvs Sig-5 R119A	CATCCCTTCCTCTCTCCACGGCGAAGAAATAGCTTCCCGTG
Fwd Sig-6	AGCAGCGCTAGCATGCAGGGAGCCCAGGAAGCC
Rvs Sig-6	AGCAGCACCGGTTCACTTGTGTATCTTGATTTC
Fwd Sig-6 R122A	CAATGCTGCATACTTCTTTGCGTTGAAGTCCAAATGGATG
Rvs Sig-6 R122A	CATCCATTTGGACTTCAACGCAAAGAAGTATGCAGCATTG
Fwd Sig-7	AGCAGCGCTAGCATGCTGCTGCTGCTGCTGCTG
Rvs Sig-7	AGCAGCACCGGTTTACTTGGGGATCTTGATCTC
Fwd Sig-7 R124A	GAGATACTTCTTTGCAATGGAGAAAGGAAATATAAAATG
Rvs Sig-7 R124A	CATTTTATATTTCCTTTCTCCATTGCAAAGAAGTATCTC
Fwd Sig-8	AGCAGCGCTAGCATGCTGCTGCTGCTGCTGCTG
Rvs Sig-8	AGCAGCACCGGTTCAGCCTCTGACTTCTTTGC
Fwd Sig-8 R125A	GATAAGGGGTCATATTTCTTTGCACTAGAGAGAGGAAG
Rvs Sig-8 R125A	CTTCCTCTCTCTAGTGCAAAGAAATATGACCCCTTATC
Fwd Sig-9	AGCAGCGCTAGCATGCTGCTGCTGCTGCTGCCC
Rvs Sig-9	AGCAGCACCGGTTCATCTGTGGATCTTGATCTC
Fwd Sig-9 R120A	GCGGGGAGATACTTCTTTGCTATGGAGAAAGGAAGTATAAAATG
Rvs Sig-9 R120A	CATTTTATACTTCCTTTCTCCATAGCAAAGAAGTATCTCCCCGC
Fwd Sig-10	AGCAGCGCTAGCATGCTACTGCCACTGCTGCTG
Rvs Sig-10	AGCAGCACCGGTTCATTGGAACTTGACTTCTGC
Fwd Sig-10 R119A	GAGTCACAGTACTTCTTTGCGGTGGAGAGAGGAAGCTATG
Rvs Sig-10 R119A	CATAGCTTCCTCTCTCCACCGCAAAGAAGTACTGTGACTC
Fwd Sig-11	AGCAGCGCTAGCATGCTGCTGCTGCCCCTGCTG
Rvs Sig-11	AGCAGCACCGGTTCACTTTGGAACCATCCCTG
Fwd Sig-11 R120A	GAGGCATGGTACTTCTTTGCGGTGGAGAGAGGAAGCCGTG
Rvs Sig-11 R120A	CACGGCTTCCTCTCTCCACCGCAAAGAAGTACCATGCCTC
Fwd Sig-15	AGCAGCGCTAGCATGGAAAAGTCCATCTGGCTG
Rvs Sig-15	AGCAGCACCGGTTCACGGTGAGCACATGGTGGC
Fwd Sig-15 R143A	GACCGCCGCTACTTCTGCGCCGTCGAGTTCGCCGGCGAC
Rvs Sig-11 R143A	GTCGCCGGCGAACTCGACGGCGCAGAAGTAGCGGCGGTC
Fwd Sig-11 K274A	GCTCTCGGCTTCAAGGCGCTGCTGCTGCTC
Rvs Sig-15 K274A	GAGCAGCAGCAGCGCTGCGAAGCCGAGAGC

Supplementary Table 3: Additional Siglec-6 mutagenesis primers.

Primer Name	Sequence
Fwd Sig-6	AGCAGCGCTAGCATGCAGGGAGCCCAGGAAGCC
Rvs Sig-6	AGCAGCACCGGTTCACTTGTGTATCTTGATTTC
Fwd C46A	CAGGAGGGTCTGGCCGTCCTCGTACCCTG
Rvs C46A	CAGGGTACGAGGACGGCCAGACCCTCCTG
Fwd E87A	CGA AGA AGT GCA GGC GGA GAC CCG GG
Rvs E87A	CCC GGG TCT CCG CCT GCA CTT CTT CG
Fwd E88A	GAA GTG CAG GAG GCG ACC CGG GGC CG
Rvs E88A	CGG CCC CGG GTC GCC TCC TGC ACT TC
Fwd R90A	GGAGGAGACCGCGGGCCGATTCCA
Rvs R90A	GAATCGGCCCGCGGTCTCCTCCTG
Fwd R92A	CCCGGGGCGCTTTCCACCTCCTCTG
Rvs R92A	CCAGAGGAGGTGGAAAGCGCCCCGGGTC
Fwd R92K	GACCCGGGGCAAATTCCACCTCCTC
Rvs R92K	GAGGAGGTGGAATTTGCCCCGGGTC
Fwd F93A	CCC GGG GCC GAG CCC ACC TCC TC
Rvs F93A	GAG GAG GTG GGC TCG GCC CCG GG
Fwd L95A	CGG GGC CGA TTC CAC GCC CTC TGG GAT
Rvs L95A	ATC CCA GAG GGC GTG GAA TCG GCC CCG
Fwd R100A	CTCTGGGATCCCGCAAGGAAGAACTGCTC
Rvs R100A	GAGCAGTTCTTCCTTGCGGGATCCCAGAG
Fwd R101A	CTCTGGGATCCCAGAGCGAAGAACTGCTC
Rvs R101A	GAGCAGTTCTTCGCTCTGGGATCCCAGAG
Fwd R109A	CCTGAGCATCGCGGATGCCCGGAG
Rvs R109A	TCCGGGCATCGCGGATGCTCAGG
Fwd R112A	CAGAGATGCCGCCAGGAGGGACAATGC
Rvs R112A	GCATTGTCCCTCCTGGCGGCATCTCTG
Fwd R113A	CAGAGATGCCCGGGCGAGGGACAATGC
Rvs R113A	GCATTGTCCCTCGCCCGGGCATCTCTG
Fwd R114A	GAGATGCCCGGAGGGCGGACAATGCTG
Rvs R114A	CAGCATTGTCCGCCCTCCGGGCATCTC
Fwd R147A	CCCTGACCCACGCGCCCAACATCTCC
Rvs R147A	GGAGATGTTGGGCGCGTGGGTCAGGG
Fwd C172A	GCCCTGGGTCGCTGAGCAGGGGAC
Rvs C172A	GTCCCCTGCTCAGCGACCCAGGGC
Fwd E173A	CCC TGG GTC TGT GCG CAG GGG ACG
Fwd E173A	CGT CCC CTG CGC ACA GAC CCA GGG
Fwd Q174A	GGG TCT GTG AGG CGG GGA CGC CCC C
Rvs Q174A	GGG GGC GTC CCC GCC TCA CAG ACC C
Fwd G175M	CTG TGA GCA GAT GAC GCC CCC CAT CTT C
Rvs G175M	GAA GAT GGG GGG CGT CAT CTG CTC ACA G

Supplementary Table 4: Lipids used in liposome formulations and their respective supplier.

Lipid	Source	Supplier
Cholesterol	NA	Sigma
20:0 DAPC	NA	Avanti
18:1-18:1 DOPC	NA	Avanti
18:0-18:1 SOPC	NA	Avanti
18:0 DSPC	NA	Avanti
16:0 DPPC	NA	Avanti
14:0 DMPC	NA	Avanti
12:0 DLPC	NA	Avanti
18:0 PEG (2000) PE	NA	Avanti
DSPE-PEG (2000) Amine	NA	Avanti
GM1	Porcine Brain	TRB Chemedica Inc.
GM2	Bovine, Semi-synthetic	Matreya
GM3	Bovine Milk	Sigma
GM4	Chicken Egg	Matreya
GD1a	Bovine, Natural	Matreya
GD1b	Bovine, Natural	Matreya
GD3	Bovine Buttermilk	Matreya
GT1b	Bovine, Natural	Matreya
GQ1b	Porcine, Natural	Matreya

Supplementary Table 5: **Liposome size as a function of formulation.**

Formulation Parameter	PEG_{45}–DSPE	Cholesterol	GM1 (Porcine Brain)	Bulk Lipid	Bulk Lipid Description	Av. Size (d)			PDI
	(mol%)					nm			
PEG_{45}–DSPE Titration	0.5	38	0	61.5	DSPC	119	±	3	0.197
	0.5	38	3	58.5	DSPC	129	±	3	0.241
	5	38	0	57	DSPC	140	±	4	0.290
	5	38	3	54	DSPC	141	±	11	0.146
GM1 Titration	0.5	38	0	61.5	DSPC	148	±	1	0.246
	0.5	38	3	58.5	DSPC	124	±	2	0.229
	0.5	38	10	51.5	DSPC	124	±	3	0.130
	0.5	38	20	41.5	DSPC	125	±	1	0.167
Cholesterol Titration	0.5	38	3	58.5	DSPC	126	±	1	0.122
	0.5	28	3	68.5	DSPC	163	±	3	0.260
	0.5	18	3	78.5	DSPC	148	±	1	0.104
	0.5	8	3	88.5	DSPC	160	±	1	0.232
Bulk Lipid Acyl Chain Length	0.5	38	3	58.5	DLPC	110	±	2	0.223
	0.5	38	3	58.5	DMPC	122	±	3	0.299
	0.5	38	3	58.5	DPPC	125	±	1	0.094
	0.5	38	3	58.5	DSPC	136	±	1	0.147
	0.5	38	3	58.5	DAPC	141	±	1	0.093
Bulk Lipid Acyl Chain Symmetry	0.5	38	3	58.5	DSPC	136	±	3	0.145
	0.5	38	3	58.5	SOPC	124	±	10	0.122
	0.5	38	3	58.5	DOPC	125	±	2	0.109
	0.5	38	3	58.5	POPC	136	±	3	0.135
	0.5	38	3	58.5	PSPC	137	±	1	0.192

Supplementary Table 6: List of antibodies used in this study.

Antibody	Supplier	Cat. No.	Label	Clone	Isotype	Dilution
anti-human CD169	Biolegend	346003	PE	7-239	Mouse IgG1, κ	1/250 (V/V)
anti-human CD22	Biolegend	302406	PE	HIB22	Mouse IgG1, κ	1/250 (V/V)
anti-human CD33	Biolegend	983904	PE	WM53	Mouse IgG1, κ	1/250 (V/V)
anti-MAG	Santa Cruz	sc-166849	PE	A-11	Mouse IgG2a, κ	1/250 (V/V)
anti-human Siglec-5	Biolegend	452003	PE	1A5	Mouse IgG1, κ	1/250 (V/V)
anti-Siglec-6	R&D Systems	FAB2859T	PE	767329	Mouse IgG2A	1/250 (V/V)
anti-Siglec-6	R&D Systems	MAB2859	Unlabeled	767329	Mouse IgG2A	1/250 (V/V)
anti-Siglec-6	R&D Systems	FAB2859G	AF488	767329	Mouse IgG2A	1/250 (V/V)
anti-Siglec-6	R&D Systems	FAB2859T	AF594	767329	Mouse IgG2A	1/250 (V/V)
anti-human CD328	Biolegend	339203	PE	6-434	Mouse IgG1, κ	1/250 (V/V)
anti-human Siglec-8	Biolegend	347103	PE	7C9	Mouse IgG1, κ	1/250 (V/V)
anti-human Siglec-9	Biolegend	351503	PE	K8	Mouse IgG1, κ	1/250 (V/V)
anti-human Siglec-10	Biolegend	347603	PE	5G6	Mouse IgG1, κ	1/250 (V/V)
anti-Siglec-11	Biolegend	681702	Unlabeled	4C4	Mouse IgG2b	1/250 (V/V)
anti-Siglec-15	Non-commercial		Unlabeled	-	Mouse IgG1	1/250 (V/V)
anti-mouse IgG1	Biolegend	406607	PE	RMG1-1	Rat IgG	1/250 (V/V)
anti-mouse IgG2b	Biolegend	406707	PE	RMG2b-1	Rat IgG, κ	1/250 (V/V)
anti-mouse IgG	Thermofischer	A-21208	AF594	Polyclonal	Donkey, IgG	1/250 (V/V)
anti-CD19	Biolegend	302219	AF488	HIB19	Mouse IgG1, κ	1/100 (V/V)
anti-CD3	Biolegend	317321	BV605	UCHT1	Mouse IgG1, κ	1/100 (V/V)
anti-CD27	Biolegend	356437	PE/Cyanine7	M-T271	Mouse IgG1, κ	1/100 (V/V)
anti-IgD	Biolegend	348207	PerCP/Cyanine5.5	IA6-2	Mouse IgG1, κ	1/100 (V/V)
anti-CD38	Biolegend	356619	BV650	HB-7	Mouse IgG1, κ	1/100 (V/V)
anti-CD22	Biolegend	302523	BV421	S-HCL-1	Mouse IgG2b, κ	1/100 (V/V)
anti-CD117 (c-Kit)	Biolegend	332203	PE	S18022G	Mouse IgG2a, κ	1/100 (V/V)

anti-FcεR1α	Biolegend	334623	BV421	AER-37	Mouse IgG2b, κ	1/100 (V/V)
anti-CD34	Biolegend	343527	BV510	581	Mouse IgG1, κ	1/100 (V/V)
anti-CD19	Biolegend	302217	APC/Cyanine7	SJ25C1	Mouse IgG1, κ	1/100 (V/V)
anti-CD14	Biolegend	301833	BV605	M5E2	Mouse IgG2a, κ	1/100 (V/V)
anti-CD3	Biolegend	317323	BV650	OKT3	Mouse IgG2a, κ	1/100 (V/V)
anti-Siglec-8	Biolegend	347111a	PE/Cyanine7	7C9	Mouse IgG1, κ	1/100 (V/V)

Supplementary Table 7: **Primers used to make Siglec-6/8 chimeras.**

Primer Name	Sequence
Fwd Sig-6	AGCAGCGCTAGCATGCAGGGAGCCCAGGAAGCC
Rvs Sig-6	AGCAGCACCGGTTCACTTGTGTATCTTGATTTC
Fwd Sig-8	AGCAGCGCTAGCATGCTGCTGCTGCTGCTGCTG
Rvs Sig-8	AGCAGCACCGGTTCAGCCTCTGACTTCTTTGC
Fwd Sig-6 $D_{1,2}$	GTGATGGCCCTGACCCATAGGCCC
Rvs Sig-6 $D_{1,2}$	GGGCCTATGGGTCAGGGCCATCAC
Fwd Sig-6 $D_{2,3}$	CCATCCAGCTCAATGTGTCCTATGCTCCACAG
Rvs Sig-6 $D_{2,3}$	CTCTGGAGAATAGGAATTGAGCTGGATGG

Supplementary Figures

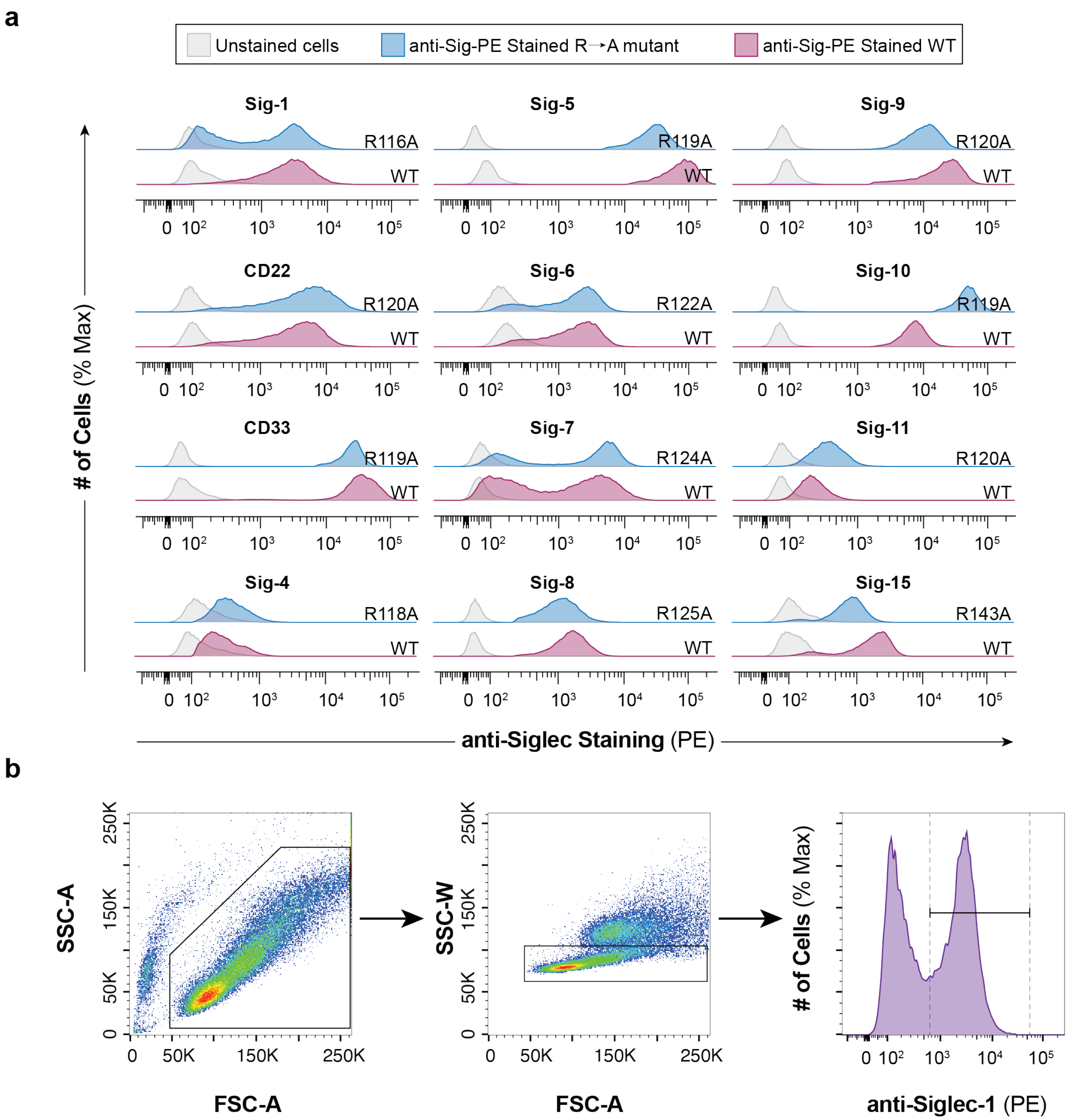

Supplementary Fig. 1: anti-Siglec-PE antibody staining, and gating strategy of CHO cell lines engineered to express each human Siglec and each corresponding arginine mutant. a, anti-Siglec-PE staining of CHO cell lines expressing each human Siglec and respective canonical arginine mutant. **b**, General gating strategy used in liposome binding experiments to isolated Siglec positive CHO cells to which liposome binding was measured.

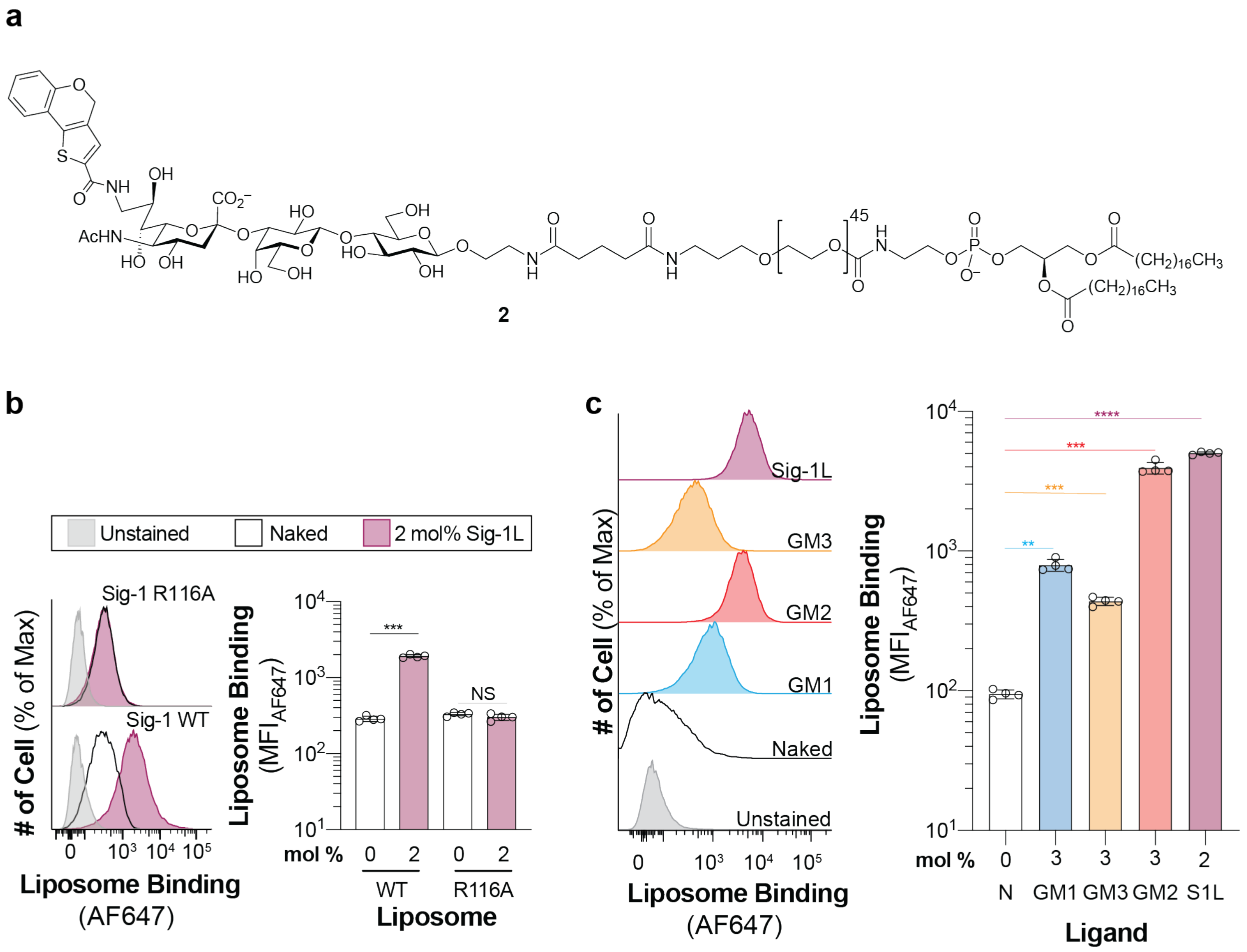

Supplementary Fig. 2: Liposomes formulated with a high affinity Siglec-1 ligand appended to PEG$_{45}$–DSPE engages WT Siglec-1 expressing CHO cells. **a**, Chemical structure of high affinity Siglec-1 ligand appended to PEG$_{45}$–DSPE (**2**). **b**, Binding of liposomes formulated with 2 mol% **2** to CHO cells expressing WT and R116A Siglec-1(n=4 technical replicates). **c**, Binding of GLLs formulated with **2** compared to GLs formulated with GM1, GM2 and GM3 (n=4 technical replicates). Data is presented with a representative flow cytometry histogram and was quantified as the mean ± one standard deviation of the median fluorescent intensity (MFI) from four technical replicates. For panels **b** and **c**, a Brown-Forsythe and Welch one-way ANOVA was used for statistical analysis. Not Significant (NS); P > 0.5; ** = 0.01 > P ≥ 0.001; *** = 0.001 > P ≥ 0.0001; **** = P < 0.0001.

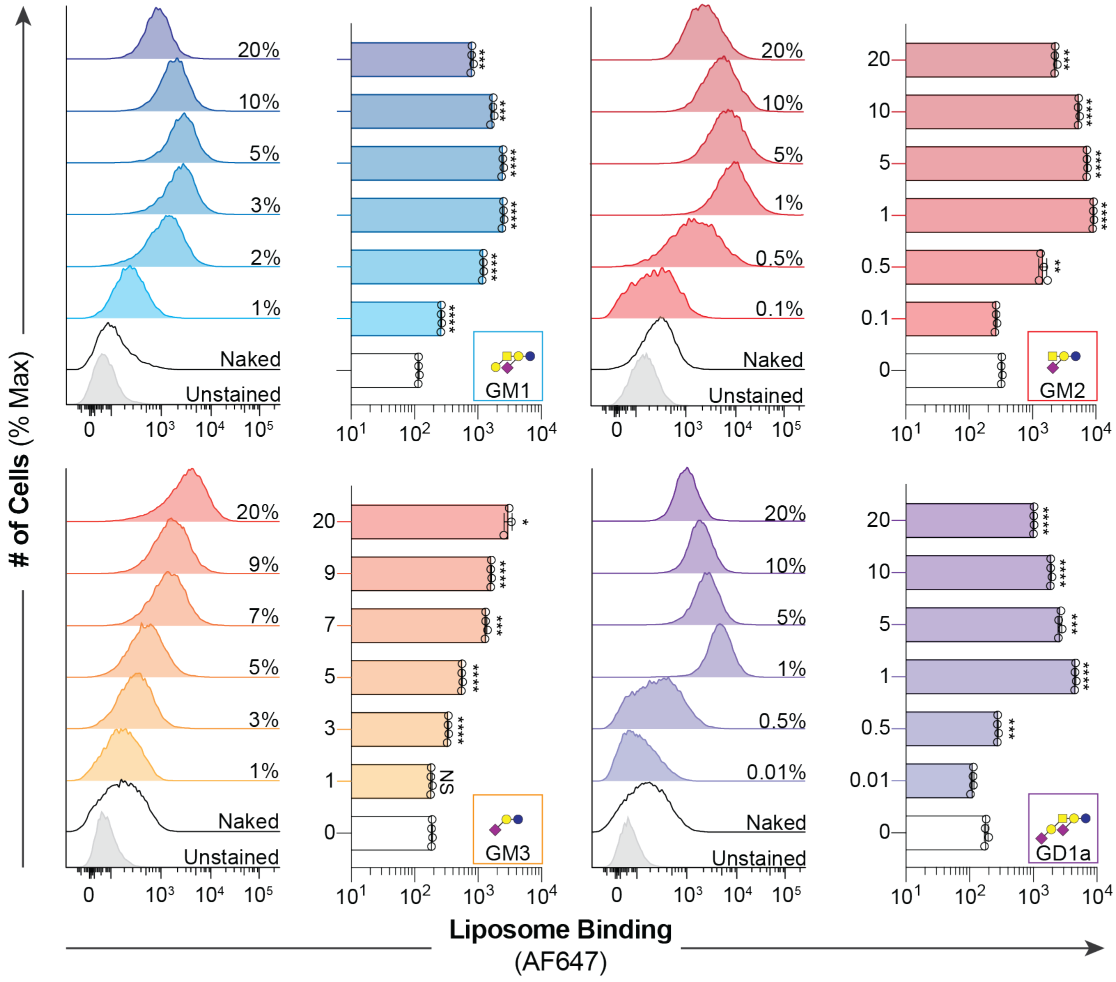

Supplementary Fig. 3: Binding of liposomes formulated with increasing ganglioside content to CHO cells expressing Siglec-1. Data is presented with a representative flow cytometry histogram and was quantified as the mean ± one standard deviation of the median fluorescent intensity (MFI) from at least three technical replicates (GM1, n=4 technical replicates; GM2, n=4 technical replicates; GM3, 4 ≥n≥ 3; GD1a, n=4 technical replicates). A Brown-Forsythe and Welch one-way ANOVA was used to compare if liposomes formulated with increasing amounts of ganglioside were significantly higher than a naked liposome. Not Significant (NS); P > 0.5; * = 0.05 > P ≥ 0.01; ** = 0.01 > P ≥ 0.001; *** = 0.001 > P ≥ 0.0001; **** = P < 0.0001.

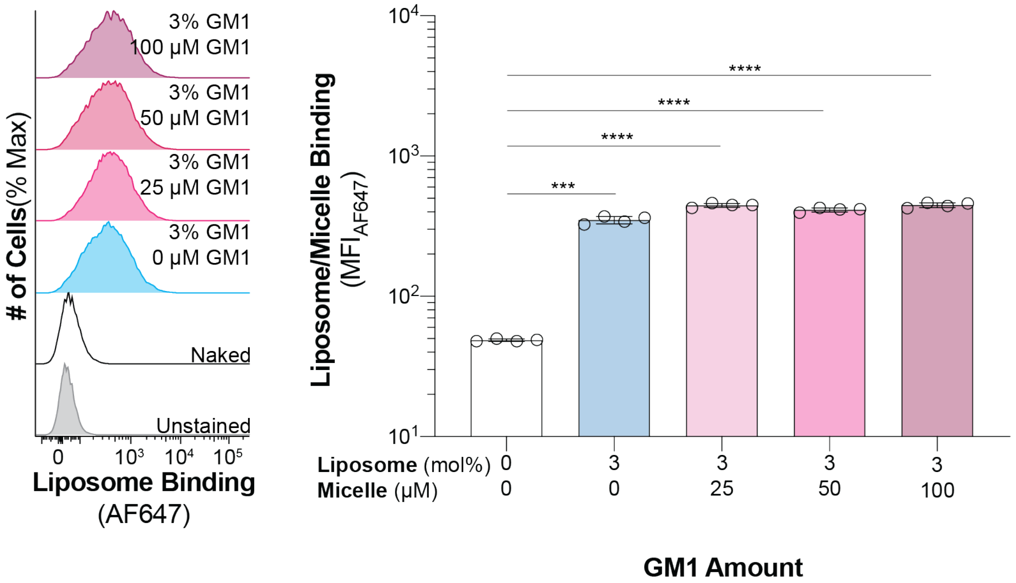

Supplementary Fig. 4: Binding of GM1 liposomes in the presence of micellular GM1 to Siglec-1 expressing CHO cells. Competition between GM1 micelles and GM1 liposomes to Siglec-1 expressing CHO cells. Data is presented with a representative flow cytometry histogram and was quantified as the mean ± one standard deviation of the median MFI from four technical replicates. A Brown-Forsythe and Welch one-way ANOVA was used to compare the binding between GM1 liposomes in the presence of GM1 micelles to naked liposomes. *** = 0.001 > P ≥ 0.0001; **** = P < 0.0001.

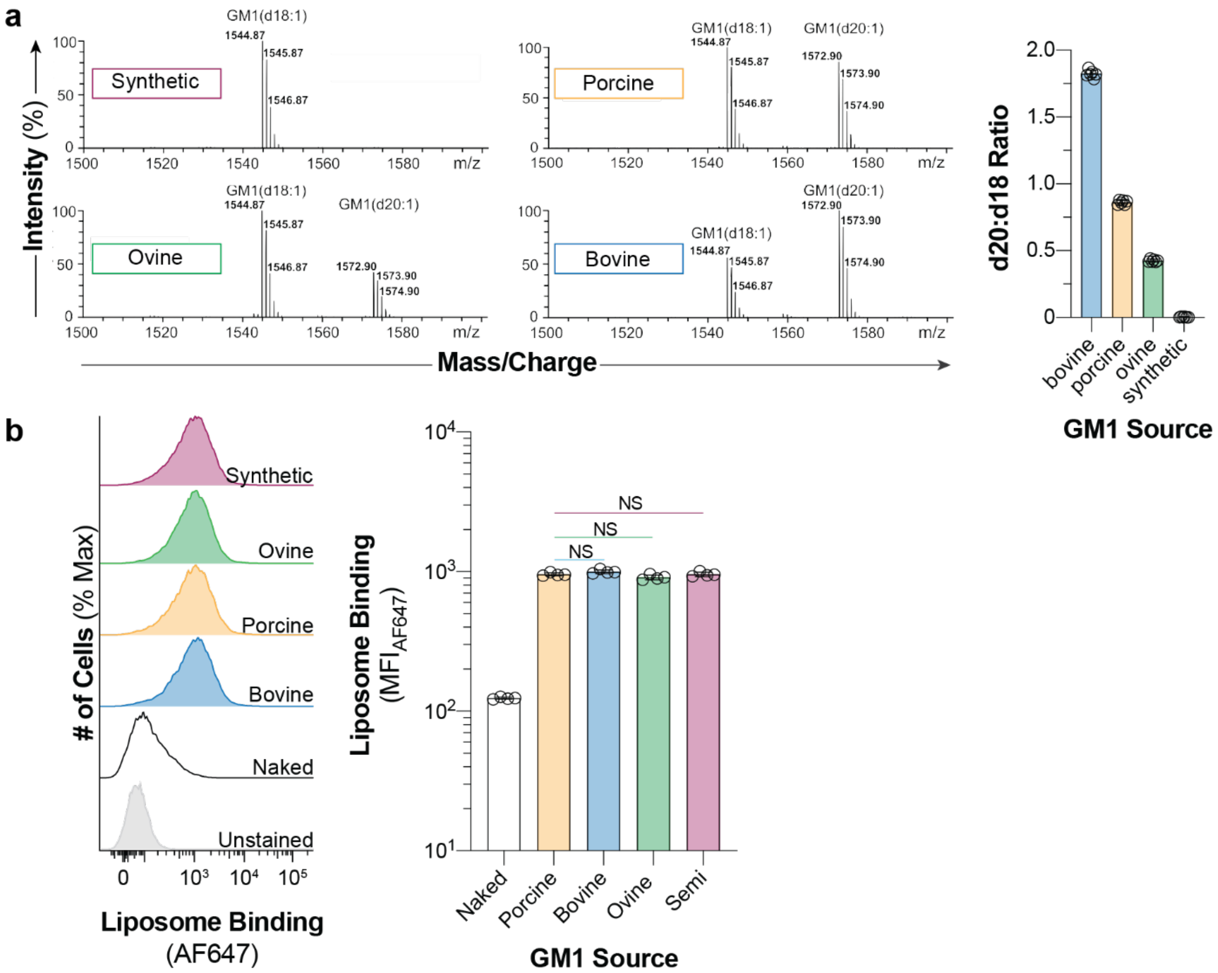

Supplementary Fig. 5: Binding of liposomes formulated with GM1 isolated from different sources to CHO cells expressing Siglec-1. **a**, Mass spectra of ganglioside GM1 isolated from different sources. Results are quantified as the mean of six technical replicates ± one standard deviation. **b**, Liposome binding of 3 mol% GM1 liposomes formulated with GM1 from different sources to Siglec-1 expressing CHO cells. Flow cytometry data is presented with a representative flow cytometry histogram and was quantified as the mean ± one standard deviation of the median fluorescent intensity (MFI) from four technical replicates. For panel **b**, a Brown-Forsythe and Welch one-way ANOVA was used for statistical analysis to compare liposomes formulated with GM1 from porcine brain (initial formulation parameter) to liposomes with GM1 from different sources. Not Significant (NS).

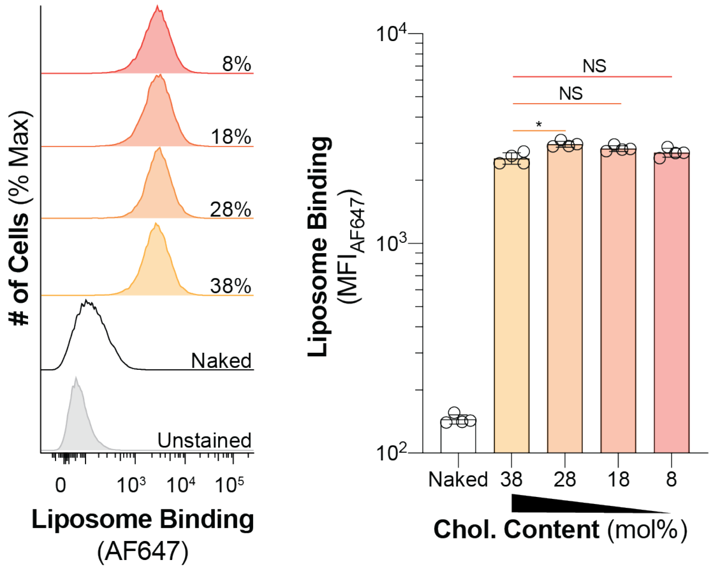

Supplementary Fig. 6: Binding of liposomes formulated with decreasing amounts of cholesterol to CHO cells expressing Siglec-1. Binding of liposome formulated with 3 mol% GM1 and varying amounts (38-8 mol%) of cholesterol to Siglec-1 expressing CHO cells. Data is presented with a representative flow cytometry histogram and was quantified as the mean ± one standard deviation of the median fluorescent intensity (MFI) from four technical replicates. A Brown-Forsythe and Welch one-way ANOVA was used for statistical analysis to compare the difference in liposome binding between 38 mol% (initial formulation parameter) to binding with the other amounts of cholesterol. Not Significant (NS); P > 0.5; * = 0.05 > P ≥ 0.01.

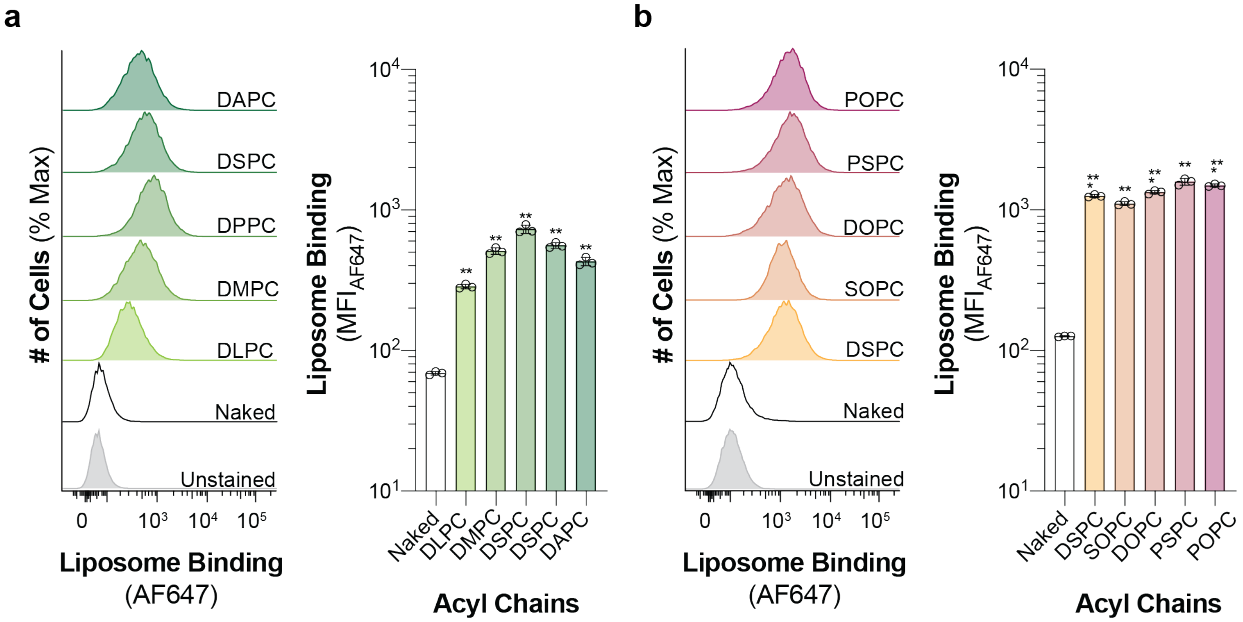

Supplementary Fig. 7: Binding of GM1 liposomes formulated with bulk lipids varying with respect to acyl chain length, symmetry, and degree of saturation to CHO cells expressing Siglec-1. **a**, Binding of 3 mol% GM1 liposomes formulated with bulk lipids with varying acyl chain lengths to Siglec-1 expressing CHO cells. **b**, Binding of 3 mol% GM1 liposomes formulated with bulk lipids with asymmetric and unsaturated acyl chains to Siglec-1 expressing CHO cells. Data is presented with a representative flow cytometry histogram and was quantified as the mean ± one standard deviation of the median fluorescent intensity (MFI) from three technical replicates. For panels **a** and **b**, a Brown-Forsythe and Welch one-way ANOVA was used to compare the binding of liposomes formulated with the various bulk lipids to that of the naked liposomes. ** = 0.01 > P ≥ 0.001; *** = 0.001 > P ≥ 0.0001.

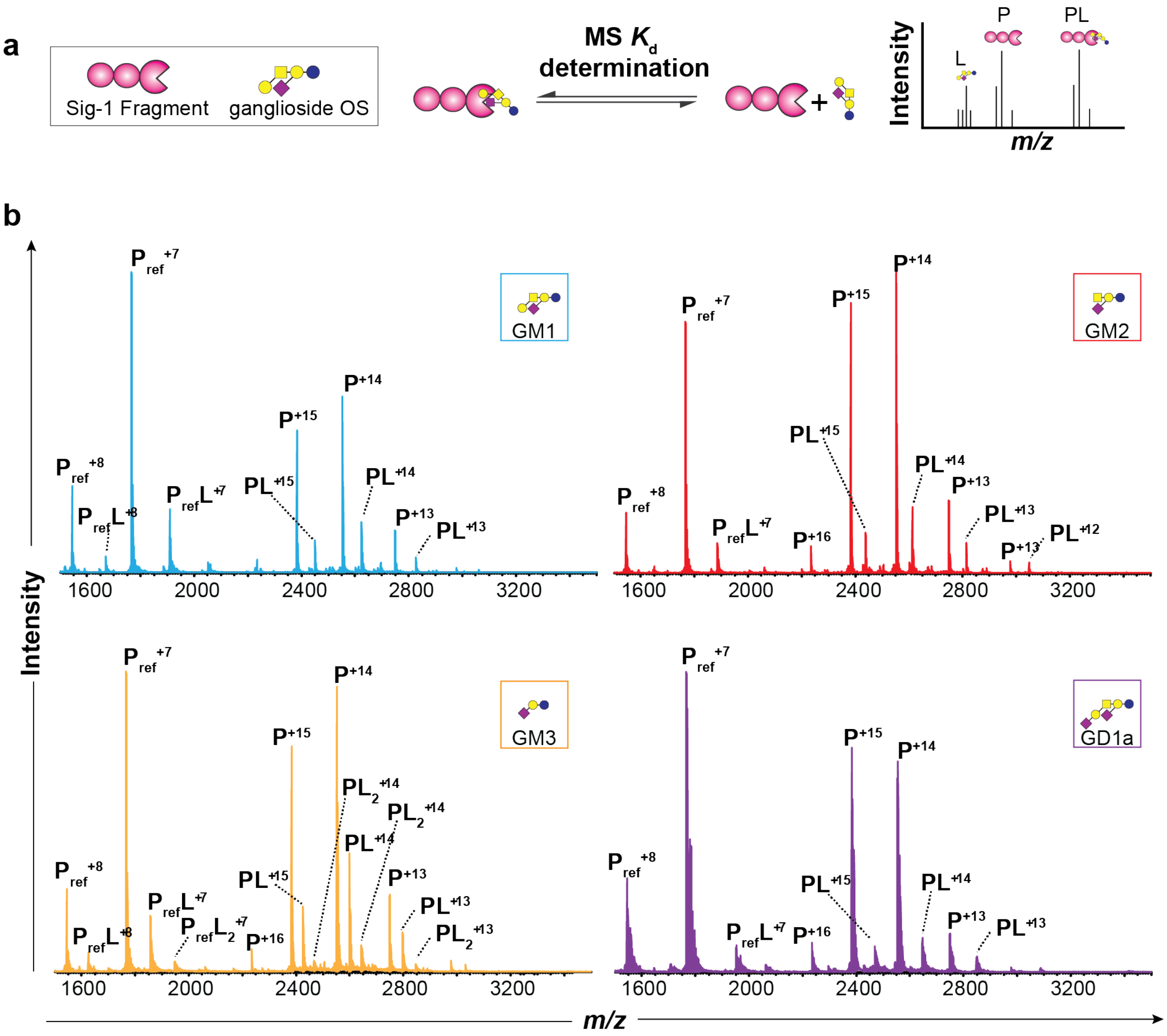

Supplementary Fig. 8: Determination of dissociation constants (K_d) between a soluble Siglec-1 fragment and the oligosaccharide of GM1, GM2, GM3, and GD1a. a, Schematic representation of the assay used to determine the K_d between Siglec-1 and the ganglioside oligosaccharide (P-protein, L-ligand, PL-protein ligand complex). **b**, Mass spectra of the soluble Siglec-1 fragment and respective ganglioside oligosaccharides. The dissociation constants were measured at a protein concentration of 3.6 µM and ganglioside oligosaccharides were titrated from 20 µM to 140 µM. Dissociation constants were calculated using equations (1) and (2).

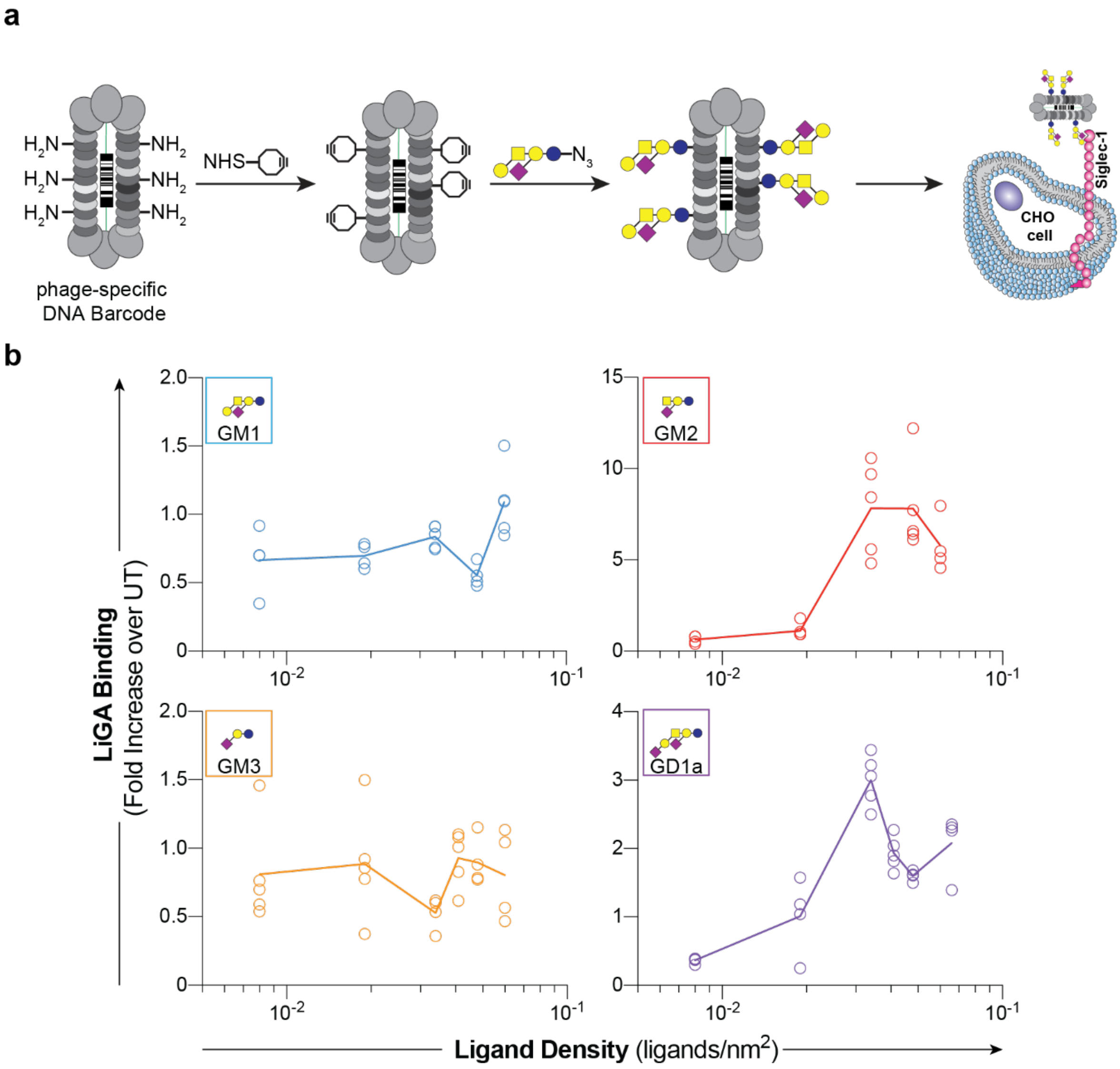

Supplementary Fig. 9: Titration of ganglioside oligosaccharide density on phage against Siglec-1 expressing CHO cells. **a**, Schematic representation of phage labelling with azide functionalized ganglioside oligosaccharides. **b**, Phage labeled with the varying densities of the oligosaccharide of GM1, GM2, GM3 and GD1a binding to CHO cells expressing Siglec-1. Binding was quantified by Next-generation sequencing and is represented by at least four technical replicates (5 ≥n≥ 4)

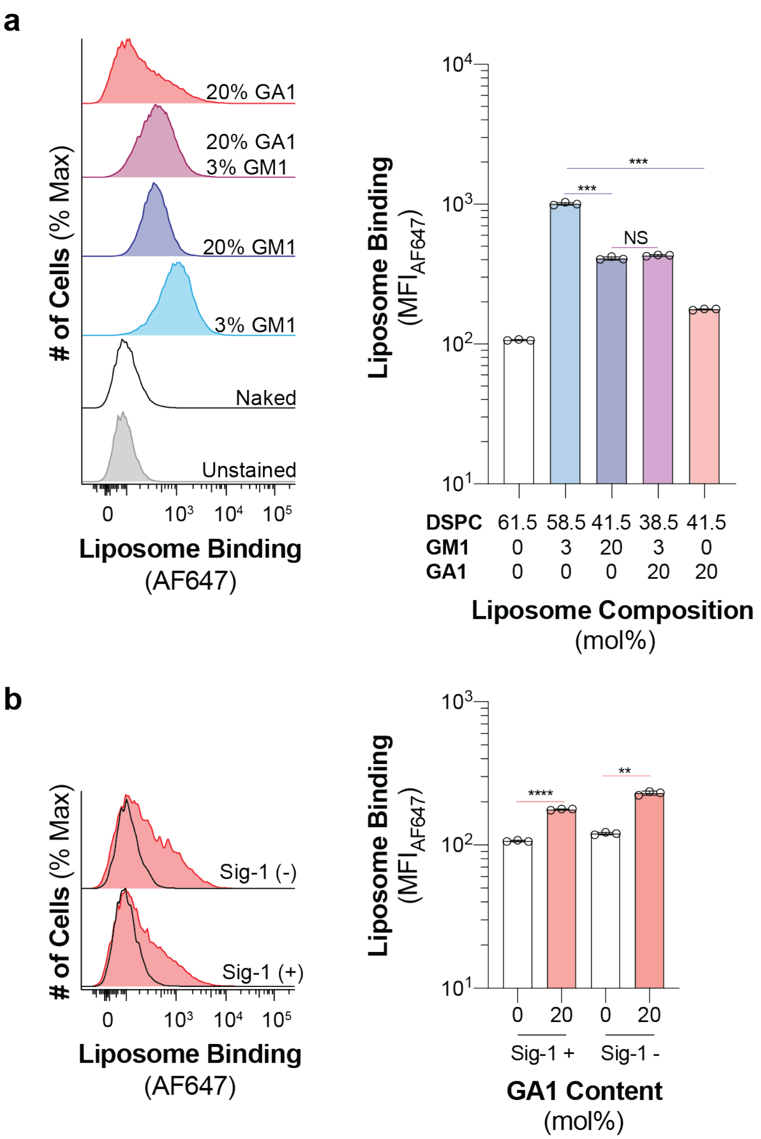

Supplementary Fig. 10: Binding of liposomes formulated with asialo-GM1 (GA1) and GM1 to CHO cells expressing Siglec-1. a, Binding of GM1 liposomes formulated with and without GA1 to Siglec-1 expressing CHO cells. **b**, Binding of 20 mol % GA1 liposomes to Siglec-1 positive and negative CHO cells. Flow cytometry data is presented with a representative flow cytometry histogram and was quantified as the mean ± one standard deviation of the median fluorescent intensity (MFI) from three technical replicates. For panels **a** and **b**, a Brown-Forsythe and Welch one-way ANOVA was used for statistical analysis. For panel **a**, statistical analysis was used to compare the effect of adding GA1 to 3 mol% GM1 liposomes on their ability to bind to Siglec-1 expressing CHO cells. For panel **b**, statistical analysis was used to determine if the minimal binding of the GA1 liposomes to the CHO cells was dependent of Siglec-1. Not Significant (NS); ** = 0.01 > P ≥ 0.001; *** = 0.001 > P ≥ 0.0001; **** = P < 0.0001.

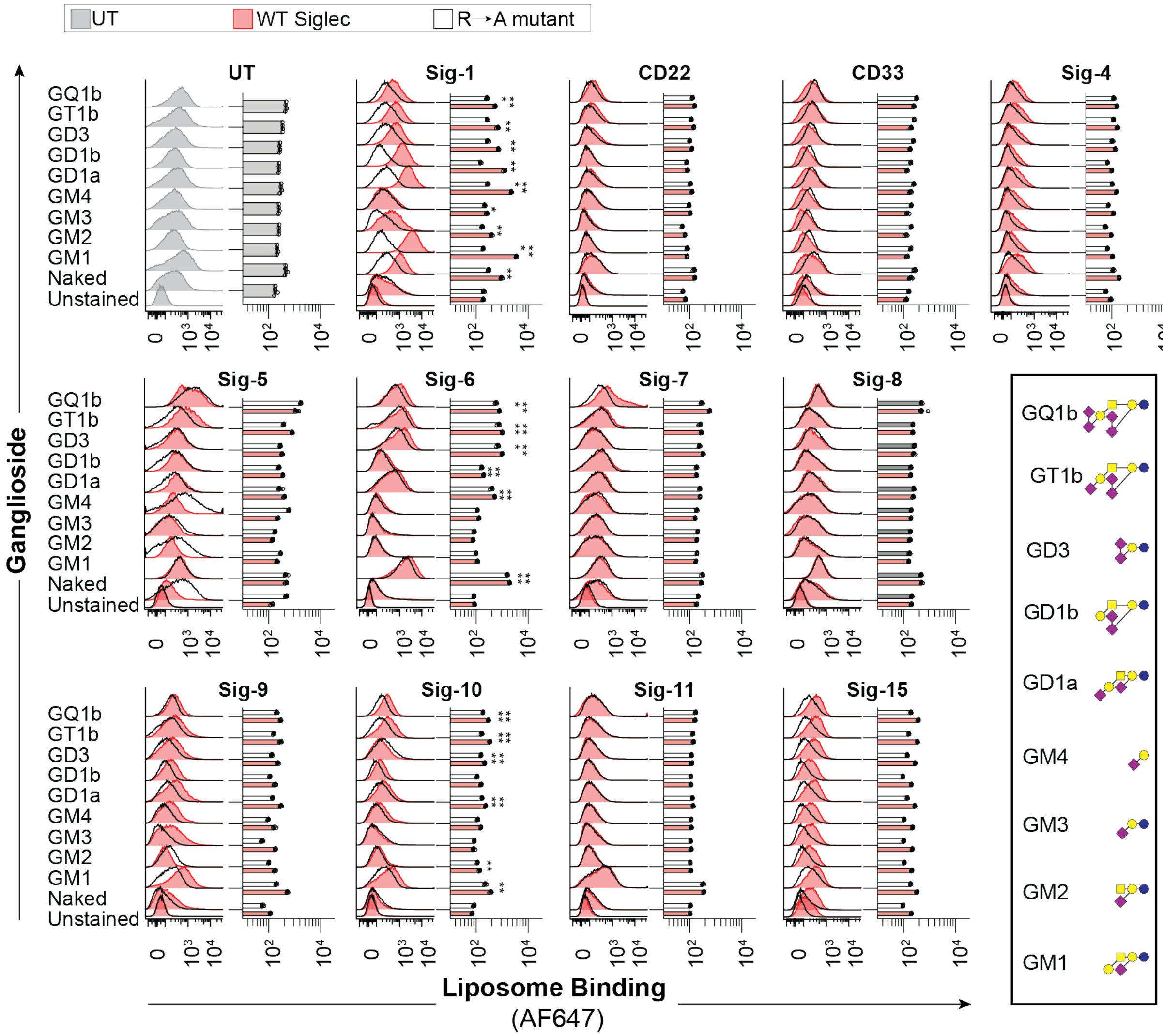

Supplementary Fig. 11: Interrogation of the human Siglec family against nine commercially available gangliosides using our optimized liposome formulation. UT-grey, WT (red), Mutant (black). Data is presented with a representative flow cytometry histogram and was quantified as the mean ± one standard deviation of the median fluorescent intensity (MFI) from at least three technical replicates (4 ≥n≥ 3). A Brown-Forsythe and Welch one-way ANOVA was used to determine if the binding of liposome formulated with a ganglioside was significantly higher than a naked liposome to CHO cells expressing WT Siglec. Not Significant (NS); P > 0.5; * = 0.05 > P ≥ 0.01; ** = 0.01 > P ≥ 0.001; *** = 0.001 > P ≥ 0.0001; **** = P < 0.0001..

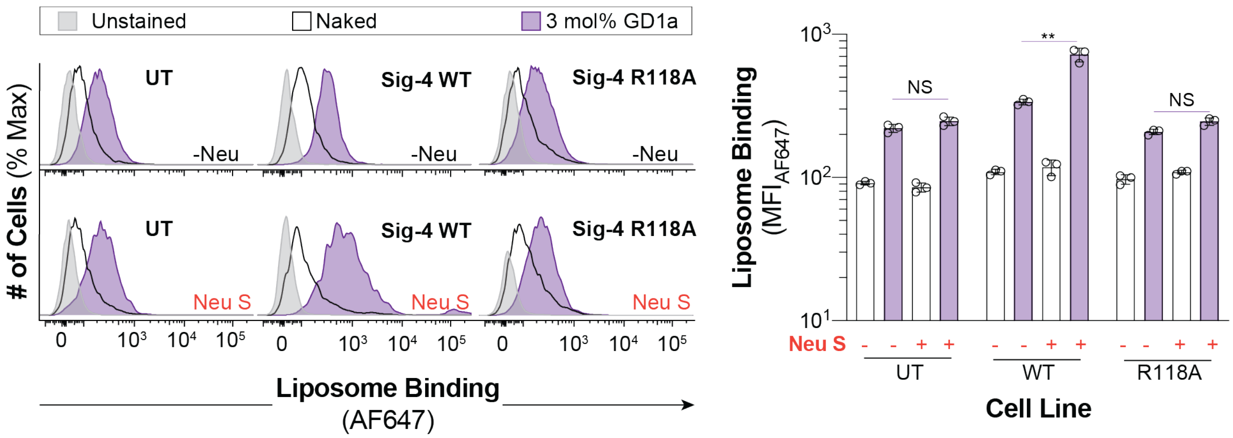

Supplementary Fig. 12: GLL binding to CHO cells expressing Siglec-4 in the absence of serum and after treatment with neuraminidase. 3 mol% GD1a GLLs binding to Siglec-4 expressing CHO cells when measured in the absence of serum glycoproteins as well as treatment with neuraminidase S. Flow cytometry data is presented with a representative flow cytometry histogram and was quantified as the mean ± one standard deviation of the median fluorescent intensity (MFI) from three technical replicates. A Brown-Forsythe and Welch one-way ANOVA was used for statistical analysis to compare the binding of 3 mol% GD1a liposome to cells before and after treatment with neuraminidase S. Not Significant (NS); ** = 0.01 > P ≥ 0.001.

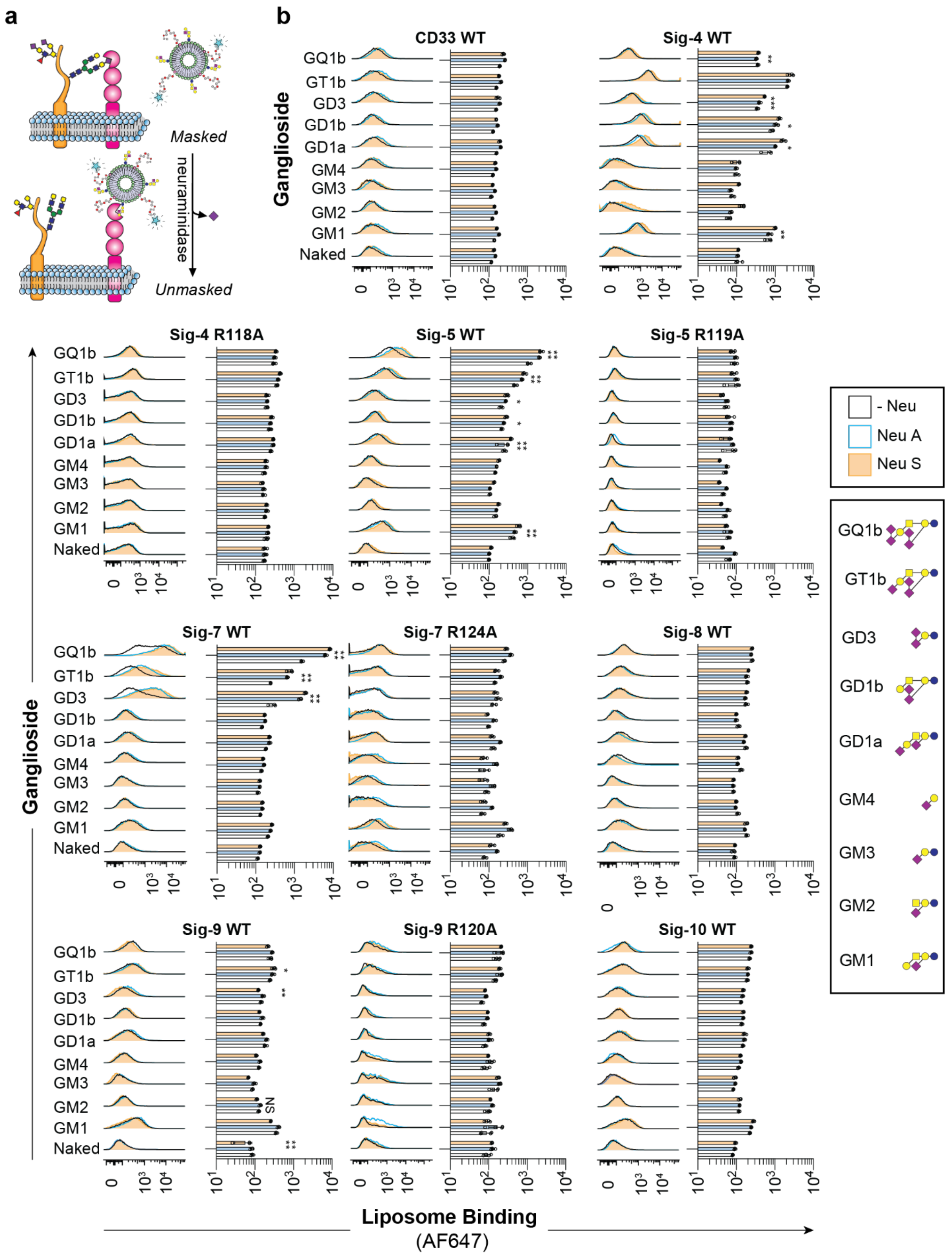
a
Masked
neuraminidase
Unmasked
b
CD33 WT
Sig-4 WT
Sig-4 R118A
Sig-5 WT
Sig-5 R119A
Sig-7 WT
Sig-7 R124A
Sig-8 WT
Sig-9 WT
Sig-9 R120A
Sig-10 WT
Ganglioside
GQ1b
GT1b
GD3
GD1b
GD1a
GM4
GM3
GM2
GM1
Naked
- Neu
Neu A
Neu S
Liposome Binding
(AF647)

Supplementary Fig. 13: Glycolipid liposome binding improves to many Siglecs when *cis* interactions are reduced by neuraminidase treatment. a, Schematic representation of the effect of neuraminidase treatment of cells on liposome binding. **b**, Binding of liposome formulated with the nine commercially available gangliosides in our optimized liposome formulation to CHO cells expressing select human Siglecs after treatment with neuraminidase A (blue) and neuraminidase S (orange). Data is presented with a representative flow cytometry histogram and was quantified as the mean ± one standard deviation of the median fluorescent intensity (MFI) from at least three technical replicates ($4 \geq n \geq 3$). A Brown-Forsythe and Welch one-way ANOVA was used to determine if the binding of liposome formulated with a ganglioside was significantly higher than a naked liposome after treatment with neuraminidase S. Not Significant (NS); $P > 0.5$; * = $0.05 > P \geq 0.01$; ** = $0.01 > P \geq 0.001$; *** = $0.001 > P \geq 0.0001$; **** = $P < 0.0001$.

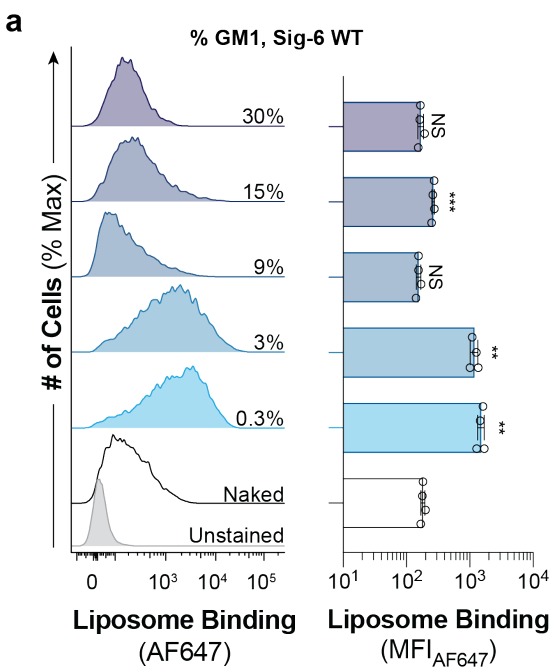

Supplementary Fig. 14: Poor binding of GLL with high ganglioside content to Siglec-6 and Siglec-7. a, **b** Binding Liposomes formulated with Increasing amounts of GM1 and GD3 to Siglec-6 and Siglec-7 expressing CHO cells respectively. For panel **b**, Siglec-7 CHO cells were treated with neuraminidase S before liposome binding. Data is presented with a representative flow cytometry histogram and was quantified as the mean ± one standard deviation of the median fluorescent intensity (MFI) from four technical replicates. For panels **a** and **b**, a Brown-Forsythe and Welch one-way ANOVA was used to compare if liposomes formulated with increasing amounts of ganglioside were significantly higher than a naked liposome. Not Significant (NS); P > 0.5; ** = 0.01 > P ≥ 0.001; *** = 0.001 > P ≥ 0.0001; **** = P < 0.0001.

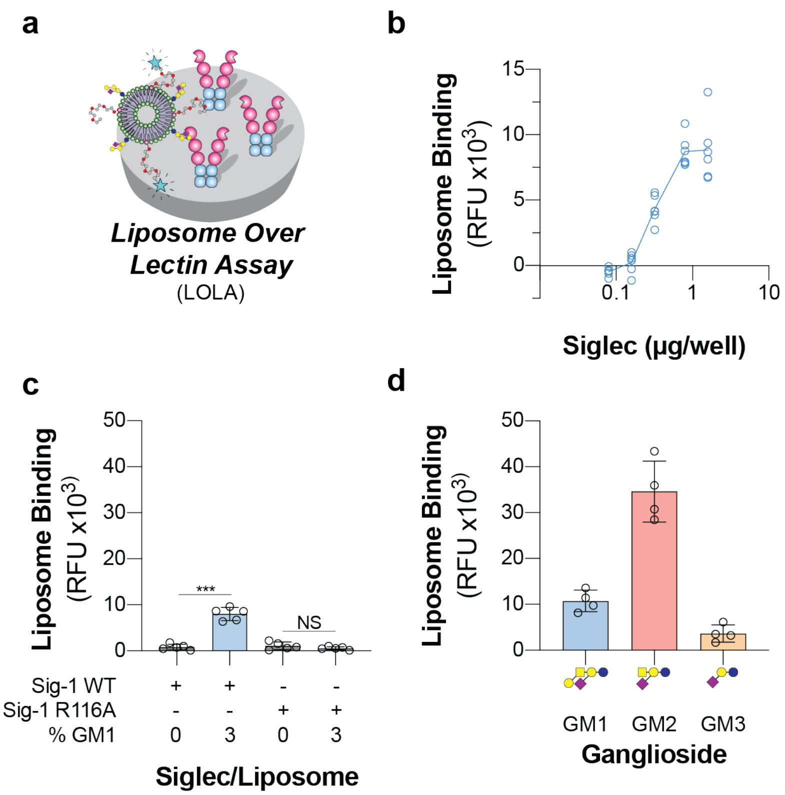

Supplementary Fig. 15: Development of Liposome Over Lectin Assay (LOLA) with Siglec-1. **a**, Schematic representation of the LOLA (n=5 technical replicates). **b**, Influence of the amount of soluble Siglec-Fc adsorbed to the microplate on binding of 3 mol% GM1 liposomes by WT Siglec-1. **b**, Binding of 3 mol% GM1 liposomes to Siglec-1 WT and R116A mutant (n=5 technical replicates). **c**, Binding of 3 mol% GM1, GM2, and GM3 liposomes to WT Siglec-1. **d**, Liposomes formulated with GM1, GM2 and GM3 binding to WT Siglec-1 in the LOLA (n=4 technical replicates). Data is presented as the mean ± one standard deviation of the background corrected relative fluorescence units (RFU) from at least four technical replicates. For panel **c**, a Brown-Forsythe and Welch one-way ANOVA was used to compare 3 mol% GM1 liposome binding to naked liposome binding to WT and R116A Siglec-1 adsorbed to the microplate. Not Significant (NS); *** = 0.001 > P ≥ 0.0001.

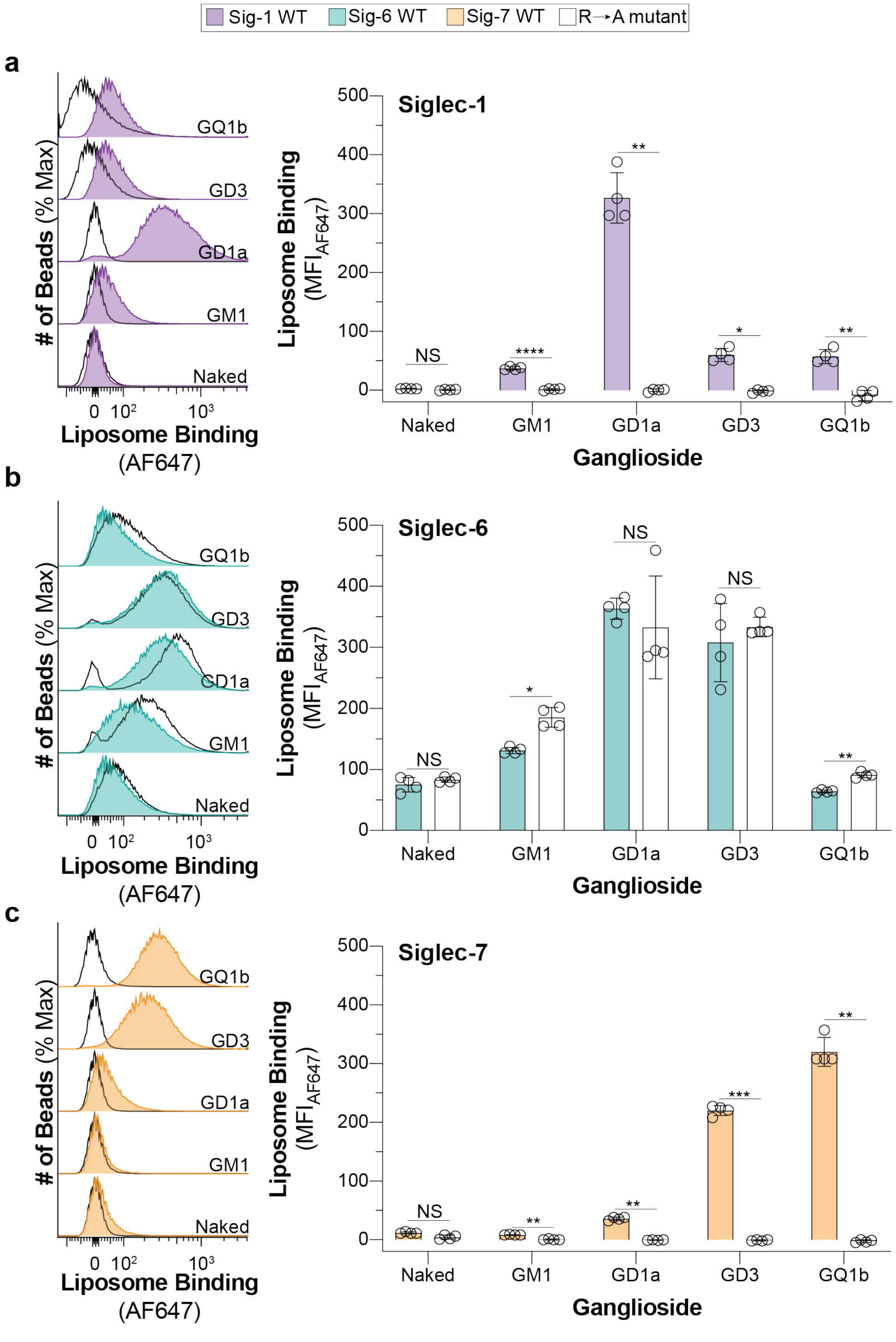

Supplementary Fig. 16: Siglec-Fc complexed with streptavidin microbeads bind GLLs. Binding of GLLs to streptavidin beads preincubated with Siglec-1 (**a**), Siglec-6 (**b**), and Siglec-7 (**c**) soluble Fc. Data is presented with a representative flow cytometry histogram and was quantified as the mean ± one standard deviation of the median fluorescent intensity (MFI) from four technical replicates. For panels **a**, **b** and **c**, a Brown-Forsythe and Welch one-way ANOVA was used to compare liposome binding between WT Siglec and its respective canonical arginine mutant. Not Significant (NS); $P > 0.5$; * = $0.05 > P \geq 0.01$; ** = $0.01 > P \geq 0.001$; *** = $0.001 > P \geq 0.0001$; **** = $P < 0.0001$.

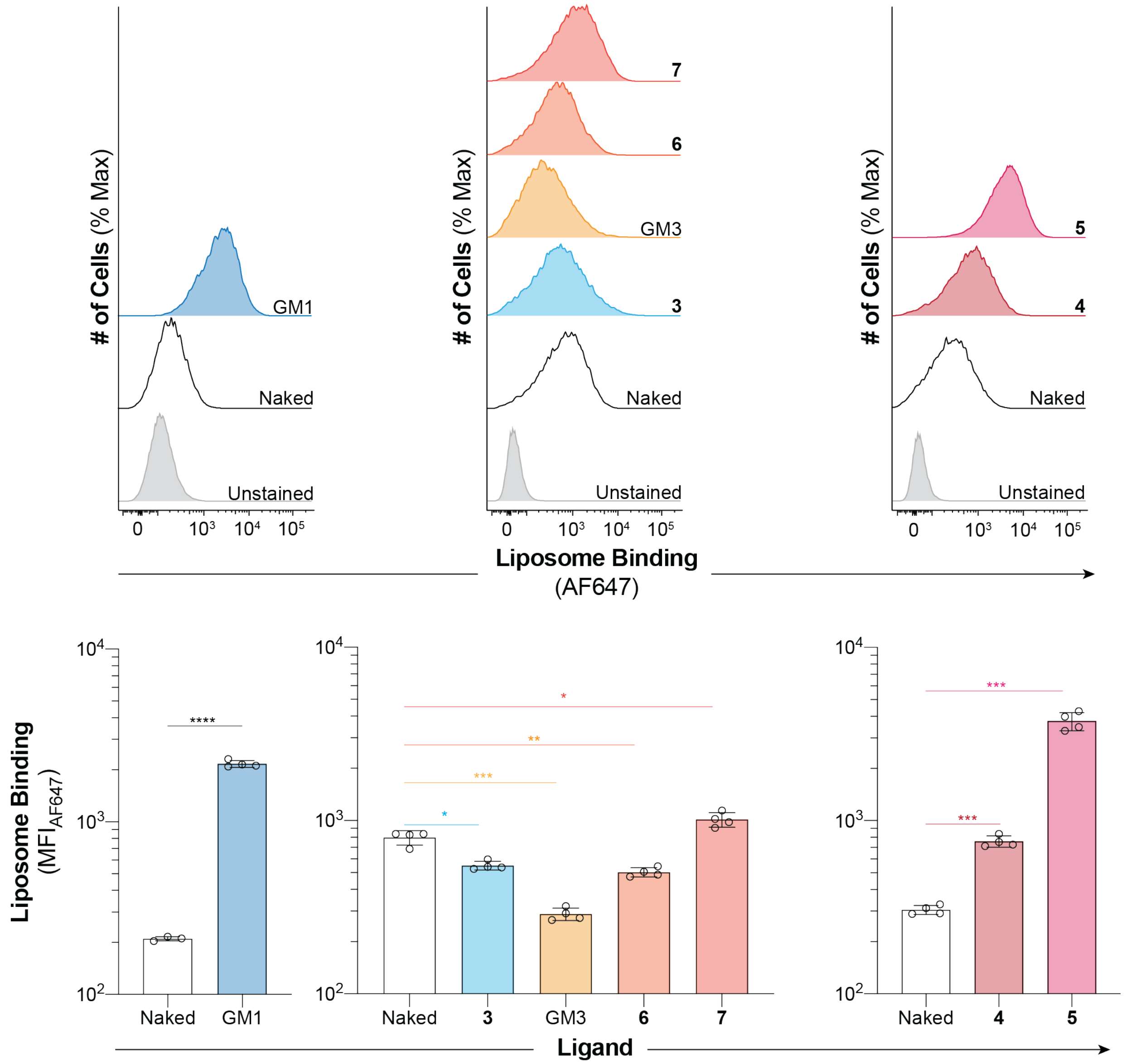

Supplementary Fig. 17: Binding of liposomes formulated with nGLs to CHO cells expressing WT Siglec-6. Liposomes formulated with 3 mol% nGLLs binding to CHO cells expressing WT Siglec-6. Data is presented with a representative flow cytometry histogram and was quantified as the mean ± one standard deviation of the median fluorescent intensity (MFI) from four technical replicates. A Brown-Forsythe and Welch one-way ANOVA was used to compare liposomes binding of liposomes formulated with various nGLLs to naked liposomes. Not Significant (NS); P > 0.5; * = 0.05 > P ≥ 0.01; ** = 0.01 > P ≥ 0.001; *** = 0.001 > P ≥ 0.0001; **** = P < 0.0001.

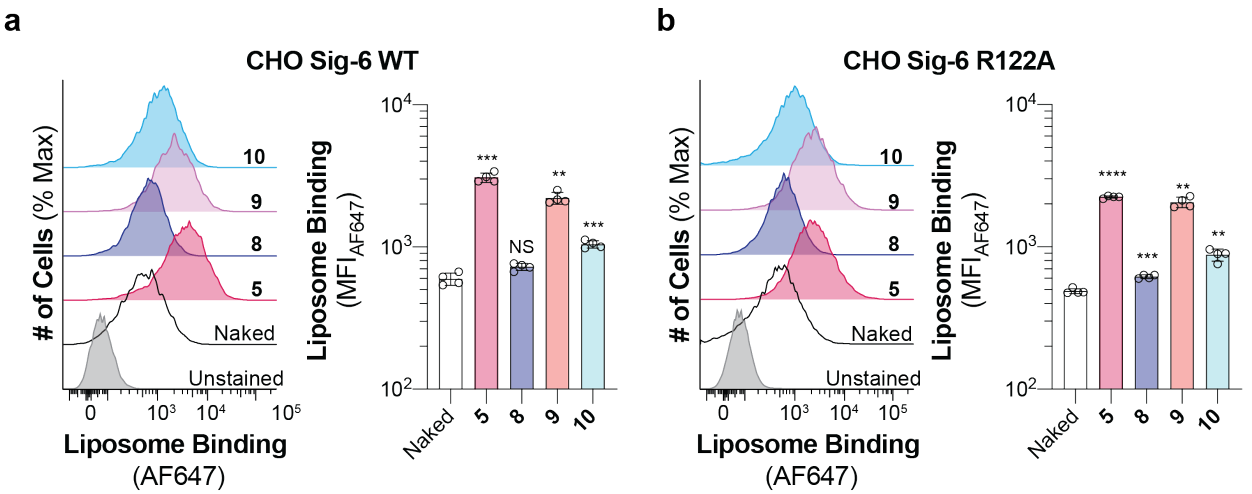

Supplementary Fig. 18: The nonconical glycolipid binding site on Siglec-6 prefers α-(2→3) over α-(2→6) sialosides and a modest preference for lactose over LacNAc scaffold. **a**, **b**, Binding liposomes formulated with 3 mol% **5**, **8**, **9** and **10** to CHO cells expressing WT and R122A Siglec-6 respectively. Data is presented with a representative flow cytometry histogram and was quantified as the mean ± one standard deviation of the median fluorescent intensity (MFI) from four technical replicates. For panels **a** and **b**, a Brown-Forsythe and Welch one-way ANOVA was used to compare the binding of liposomes formulated with nGLs to naked liposomes. Not Significant (NS); $P > 0.5$; ** = $0.01 > P \geq 0.001$; *** = $0.001 > P \geq 0.0001$; **** = $P < 0.0001$.

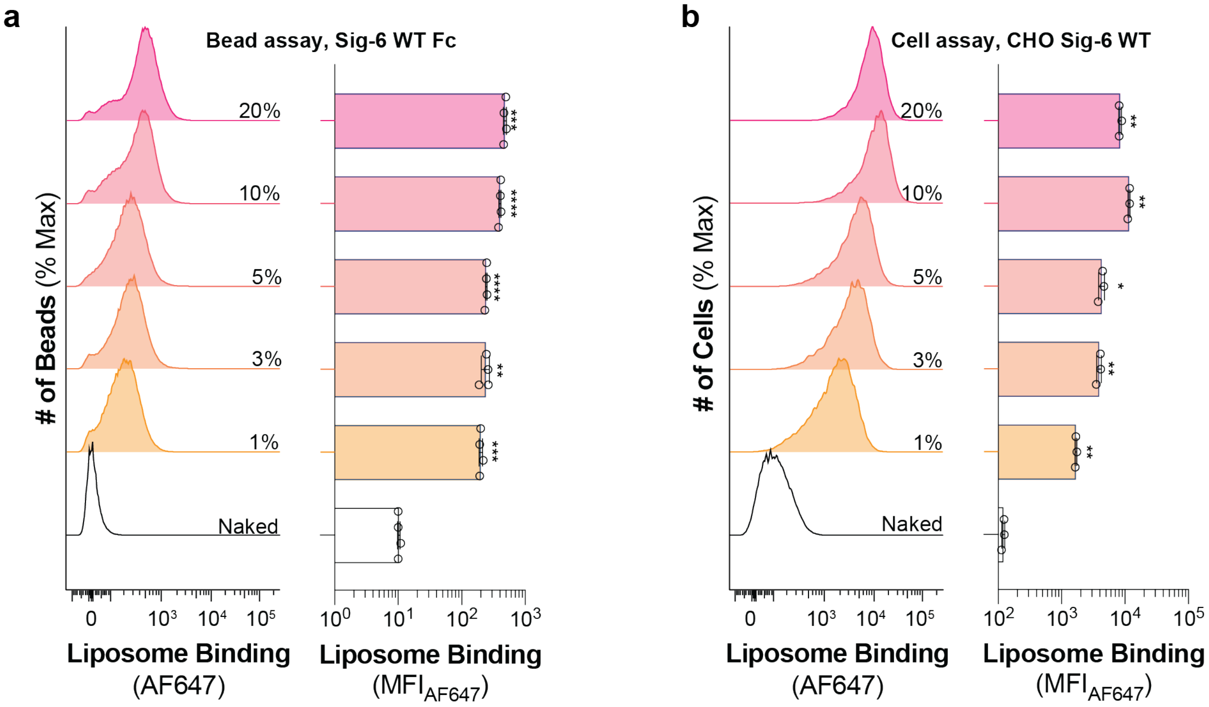

Supplementary Fig. 19: Optimizing the mol% of 5 in nGLLs for engaging Siglec-6. **a**, Titration of **5** in liposomes in the bead assay against streptavidin bead complexed with recombinant WT Siglec-6-Fc. **b**, Titration of **5** in liposomes in the cell assay against CHO cells expressing WT Siglec-6. Data is presented with a representative flow cytometry histogram and was quantified as the mean ± one standard deviation of the median fluorescent intensity (MFI) from four technical replicates. For panels **a** and **b**, a Brown-Forsythe and Welch one-way ANOVA was used to compare the binding of liposomes formulated with nGLLs to naked liposomes. $P > 0.5$; * = $0.05 > P \geq 0.01$; ** = $0.01 > P \geq 0.001$; *** = $0.001 > P \geq 0.0001$; **** = $P < 0.0001$.

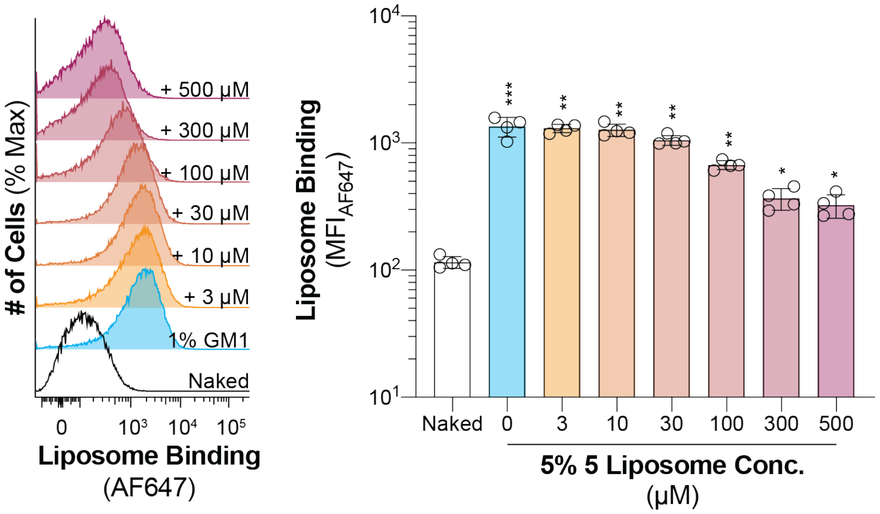

Supplementary Fig. 20: Binding competition between GM1 GLLs and 5 nGLLs against WT Siglec-6. Binding of 1 mol% GM1 liposomes to WT Siglec-6 in the presence of an increasing concentration of 5 mol% **5** nGLLs. Data is presented with a representative flow cytometry histogram and was quantified as the mean ± one standard deviation of the median fluorescent intensity (MFI) from four three technical replicates. A Brown-Forsythe and Welch one-way ANOVA was used to compare the binding of liposomes formulated with nGLLs to naked liposomes. P > 0.5; * = 0.05 > P ≥ 0.01; ** = 0.01 > P ≥ 0.001; *** = 0.001 > P ≥ 0.0001.

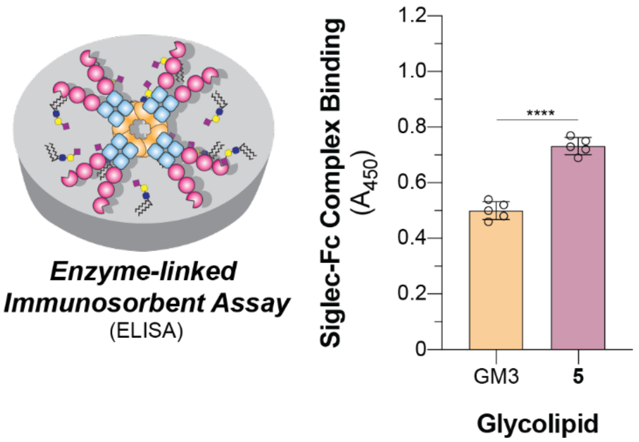

Supplementary Fig. 21: Comparison of the binding of Siglec-6-Fc complex to GM3 and nGL 5 outside of a lipid bilayer. Data is represented as the mean ± one standard deviation of five technical replicants of the background corrected A_{450nm}. The background was measured using an ethanol vehicle control. A two tailed Student's t-test was used for statistical analysis. **** = $P < 0.0001$..

a

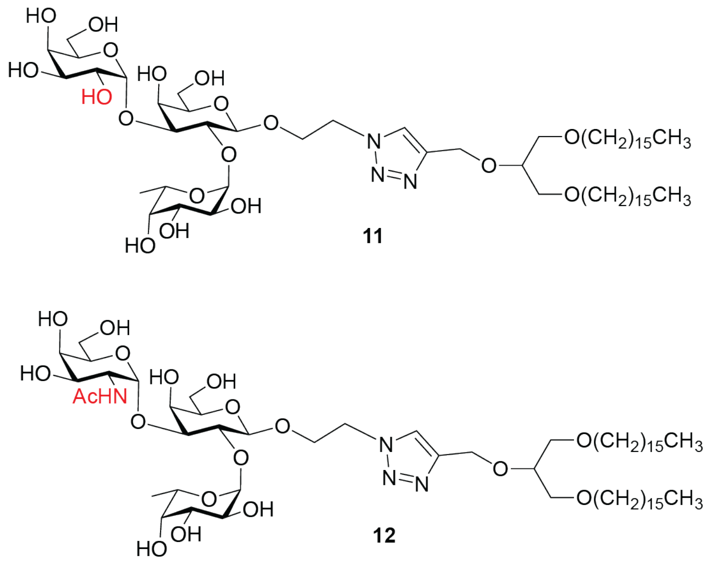

b

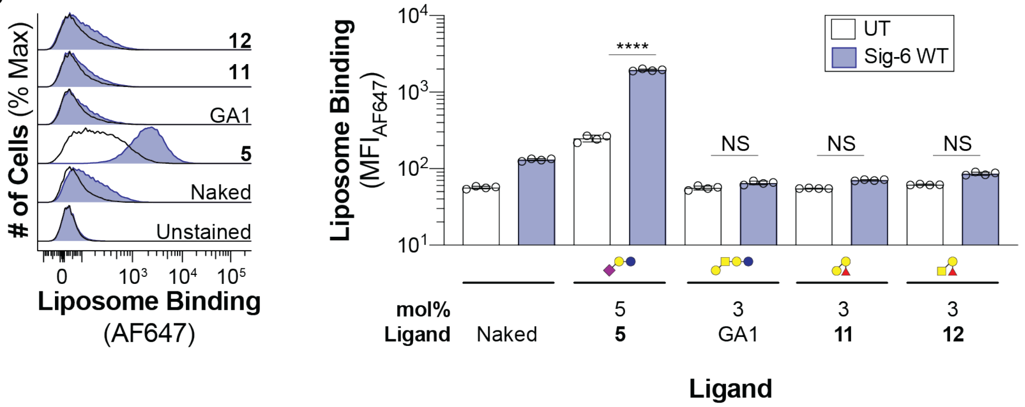

Supplementary Fig. 22: Siglec-6 does not engage nGLLs formulated with asialo neoglycolipids. **a**, Chemical structures of nGLs **11** and **12**. **b**, Liposome binding of **5**, GA1, **11**, and **12** to UT and WT Siglec-6 CHO cells. Data is presented with a representative flow cytometry histogram and was quantified as the mean ± one standard deviation of the median fluorescent intensity (MFI) from four technical replicates. For panel **b**, a Brown-Forsythe and Welch one-way ANOVA was used to compare binding of each liposome formulated with each glycolipid to untransfected CHO cells and CHO cells expressing WT Siglec-6. Not Significant (NS); **** = P < 0.0001.

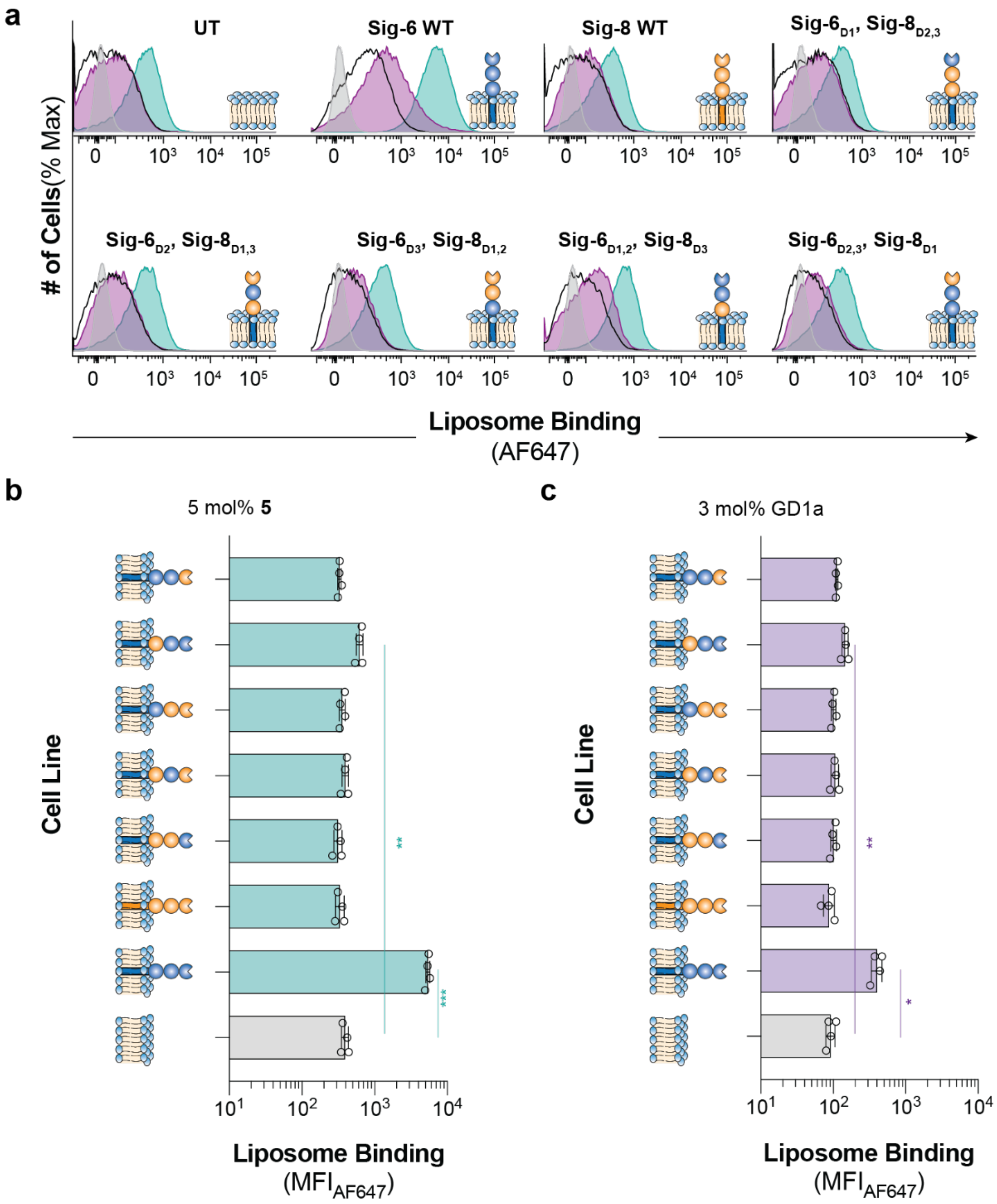

Supplementary Fig. 23: The V-set domain and first C2 domain of Siglec-6 are both required to bind glycolipids. a, Representative flow cytometry histograms of **5** and GD1a liposomes binding to Siglec-6/8 chimeras. **b**, Binding of liposomes formulated with 5 mol% **5** to CHO cells expressing each Siglec-6/8 chimera. **c**, Binding of liposomes were formulated with 3 mol% GD1a to CHO cells expressing each Siglec-6/8 chimera. For panels **b** and **c**, data was quantified as the mean ± one standard deviation of the median fluorescent intensity (MFI) from four technical replicates. a Brown-Forsythe and Welch one-way ANOVA was used to compare liposome binding to CHO cells expressing WT Siglec-6 and Siglec-$6_{D1,2}$, Sig-8_{D3} to UT CHO cells. P > 0.5; * = 0.05 > P ≥ 0.01; ** = 0.01 > P ≥ 0.001; *** = 0.001 > P ≥ 0.0001.

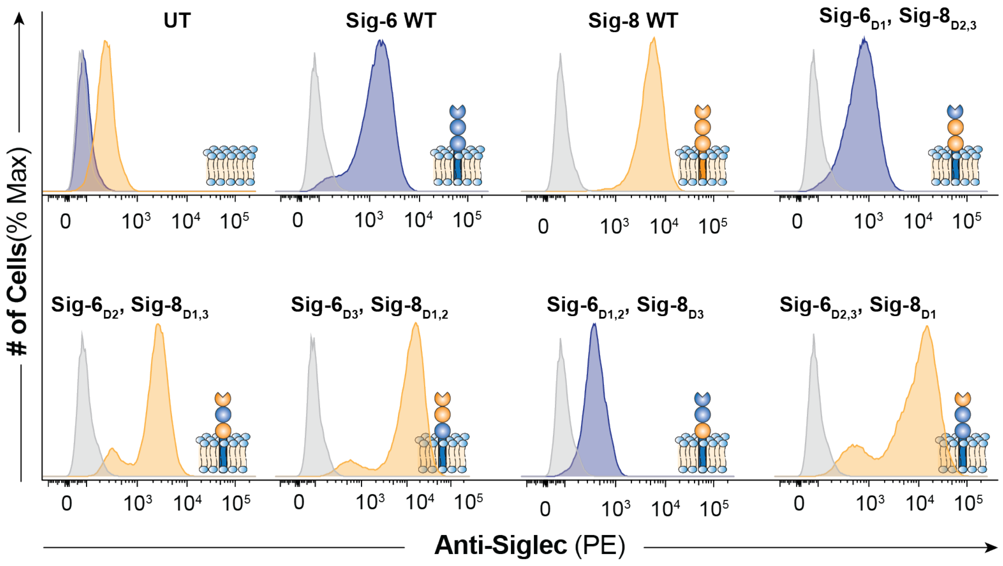

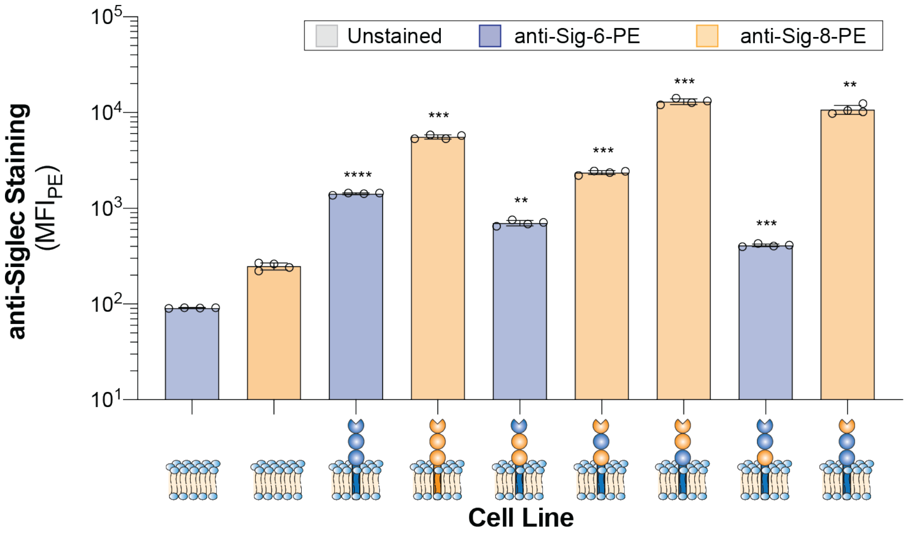

Supplementary Fig. 24: Expression levels of Siglec-6/8 chimeras on CHO cells. Representative flow cytometry histograms of antibody staining to Siglec-6/8 chimeras. Data is presented with a representative flow cytometry histogram and was quantified as the mean ± one standard deviation of the median fluorescent intensity (MFI) from four technical replicates. A Brown-Forsythe and Welch one-way ANOVA was used to compare the level of anti-Siglec-6/8 staining of each chimera to untransfected CHO cells. ** = 0.01 > P ≥ 0.001; *** = 0.001 > P ≥ 0.0001; **** = P < 0.0001.

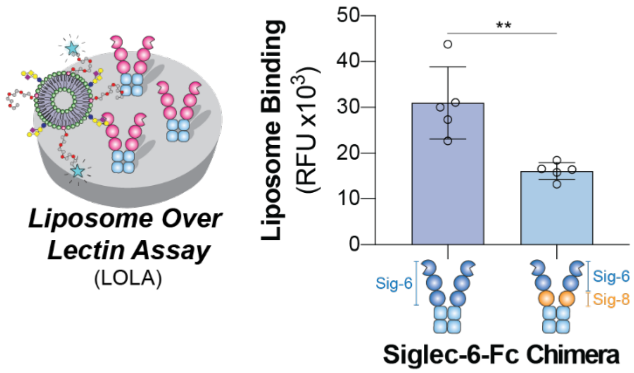

Supplementary Fig. 25: A chimera of Siglec-6/8 containing the first two extracellular domains of Siglec-6 and the third extracellular domain of Siglec-8 shows significant binding in the LOLA. Data is presented as the mean of five technical replicates ± one standard deviation of the background subtracted (naked liposome) relative fluorescence units (RFU). A two tailed Student's t-test was used for statistical analysis. ** = $0.01 > P \geq 0.001$;

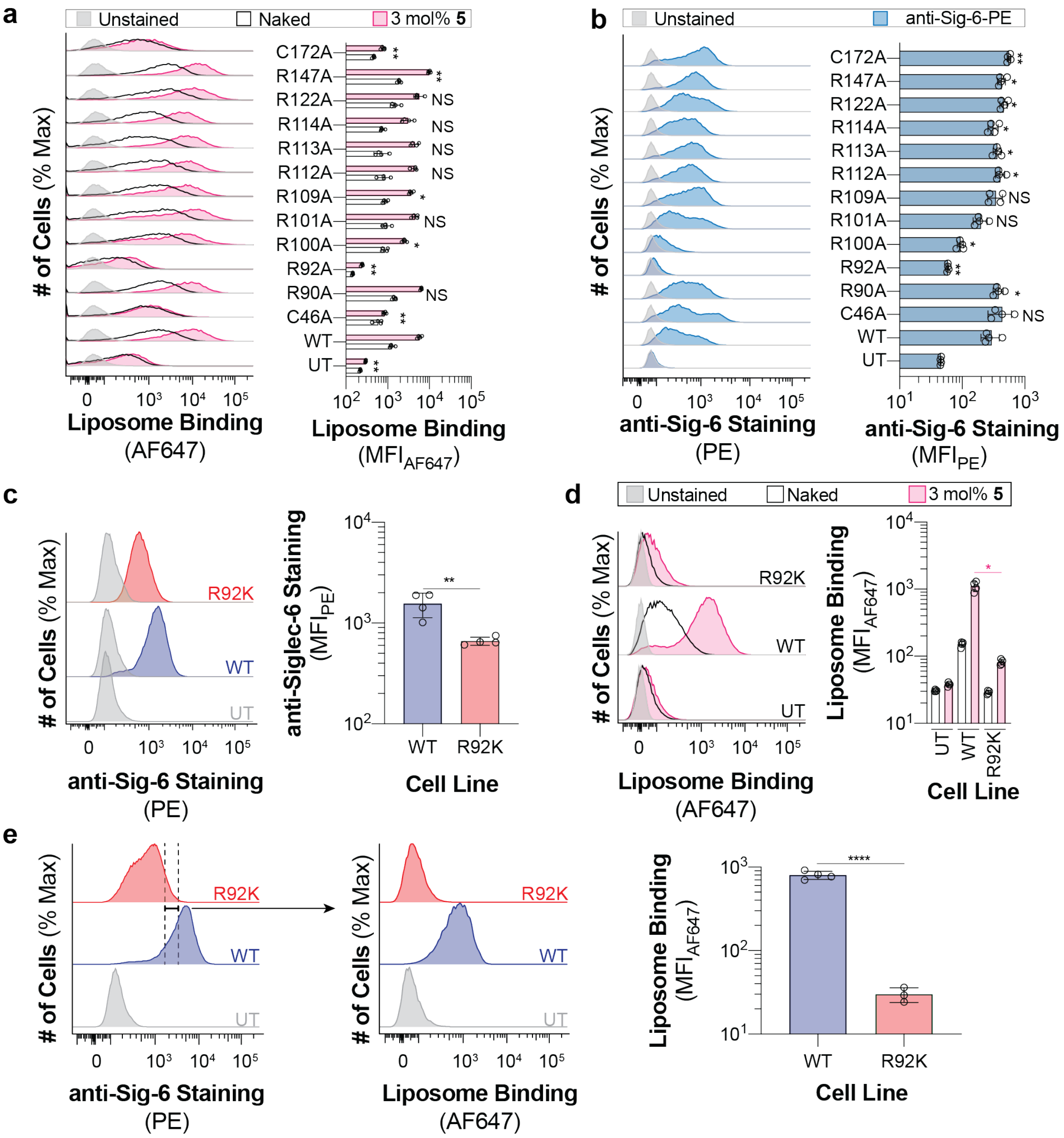

Supplementary Fig. 26: Analysis of nGLL binding to Siglec-6 mutants on CHO cells. a, **5** liposome binding to CHO cells expressing different mutants (R→A and C→A) of Siglec-6 (n=4 technical replicates). **b**, Siglec-6 expression levels on CHO cells expressing different mutants (R→A and C→A) of Siglec-6 (n=4 technical replicates). **c**, Siglec-6 expression levels on CHO cells expressing R92K Siglec-6 (n=4 technical replicates). **d**, **5** liposome binding to CHO cells expressing R92K Siglec-6 (n=4 technical replicates). **e**, **5** liposome binding to WT Siglec-6 and R92K Siglec-6 gated on identical Siglec-6 expression levels as defined by the gate (4≥n≥3 technical replicates). For all panels, data is presented with a representative flow cytometry histogram and was quantified as the mean ± one standard deviation of the median fluorescent intensity (MFI) from at least three technical replicates. A Brown-Forsythe and Welch one-way ANOVA was used for statistical analysis in panels **a**, **b,** and **d**. For panel **a**, statistical analysis was used to determine if the **5** liposome binding from was different between the WT and each mutant. For panel **b**, statistical analysis was used to determine if the anti-Siglec-6-PE staining was different compared to UT CHO cells. For panel **d**, statistical comparison was between the **5** liposome binding between WT Siglec-6 and R92K Siglec-6 CHO cells. For panels **c** and **e**, a two tailed Student's t-test was used for statistical analysis. Not Significant (NS); P > 0.5; * = 0.05 > P ≥ 0.01; ** = 0.01 > P ≥ 0.001; *** = 0.001 > P ≥ 0.0001; **** = P < 0.0001.

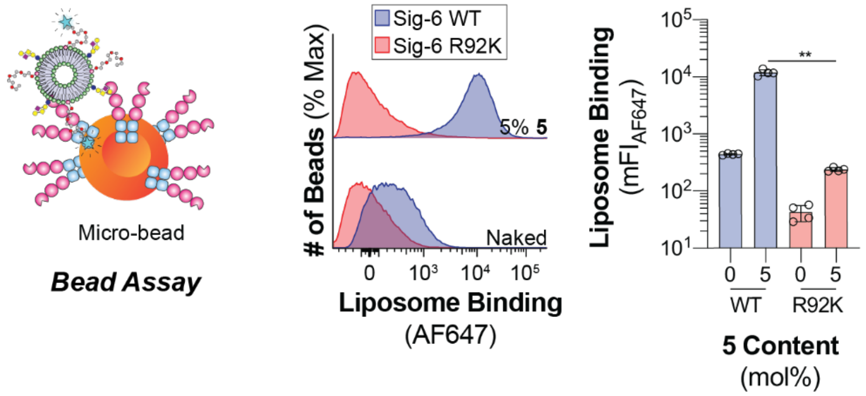

Supplementary Fig. 27: Binding of 5 nGLLs to WT and R92K Siglec-6-Fc in the bead assay. Data is presented with a representative flow cytometry histogram and was quantified as the mean ± one standard deviation of the mean fluorescent intensity (mFI) from four technical replicates. A Brown-Forsythe and Welch one-way ANOVA was used to compare the binding of 5% **5** nGLLs between WT and R92K Siglec-6. ** = $0.01 > P \geq 0.001$.

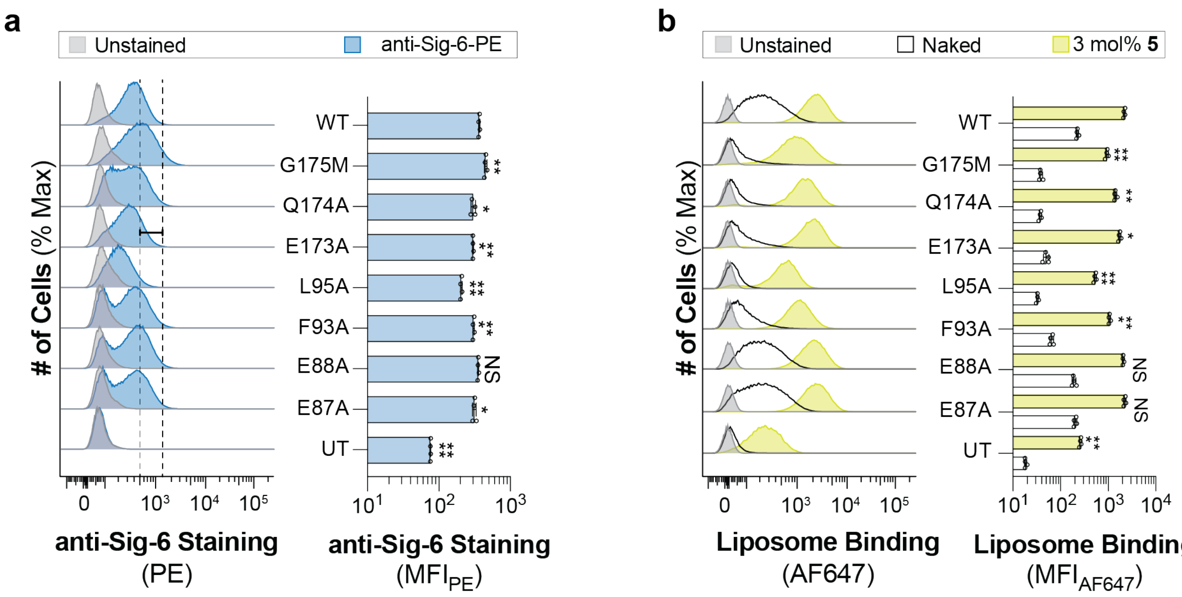

Supplementary Fig. 28. Binding of liposomes formulated with 5% 5 to mutants of residues that reside at the interface of the V-set and first C2 domain of Siglec-6 on CHO cells. a, Staining of Siglec-6 mutants with anti-Siglec-6-PE. **b**, **5** liposome binding to CHO cells expressing different mutants of Siglec-6. Data is presented with a representative flow cytometry histogram and was quantified as the mean ± one standard deviation of the median fluorescent intensity (MFI) of four technical replicates. For panel **a**, a Brown-Forsythe and Welch one-way ANOVA was used to determine if the anti-Siglec-6-PE staining was different between the WT and each mutant. For panel **b**, a Brown-Forsythe and Welch one-way ANOVA was used to determine if the **5** liposome binding from was different between the WT and each mutant. Not Significant (NS); P > 0.5; * = 0.05 > P ≥ 0.01; ** = 0.01 > P ≥ 0.001; *** = 0.001 > P ≥ 0.0001; **** = P < 0.0001.

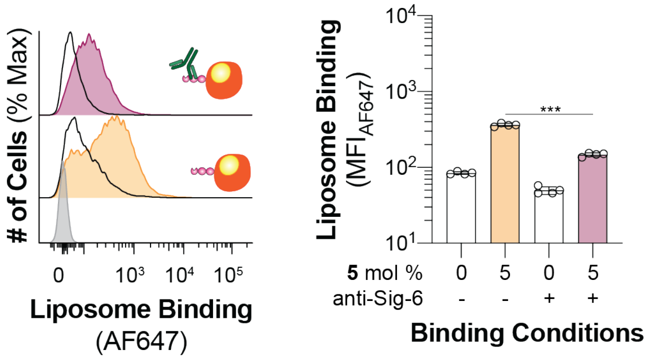

Supplementary Fig. 29: Blocking nGLL binding to Siglec-6 expressing CHO cells with anti-Siglec-6 antibody. CHO cells expressing Siglec-6 were pre-incubated with anti-Siglec-6 antibody prior to incubation with **5** nGLLs followed by flow cytometry. Data is presented with a representative flow cytometry histogram and was quantified as the mean ± one standard deviation of the median fluorescent intensity (MFI) from four technical replicates. A Brown-Forsythe and Welch one-way ANOVA was used to compare **5** liposome binding before and after treatment with the anti-Siglec-6 antibody to Siglec-6 expressing CHO cells. *** = $0.001 > P \geq 0.0001$.

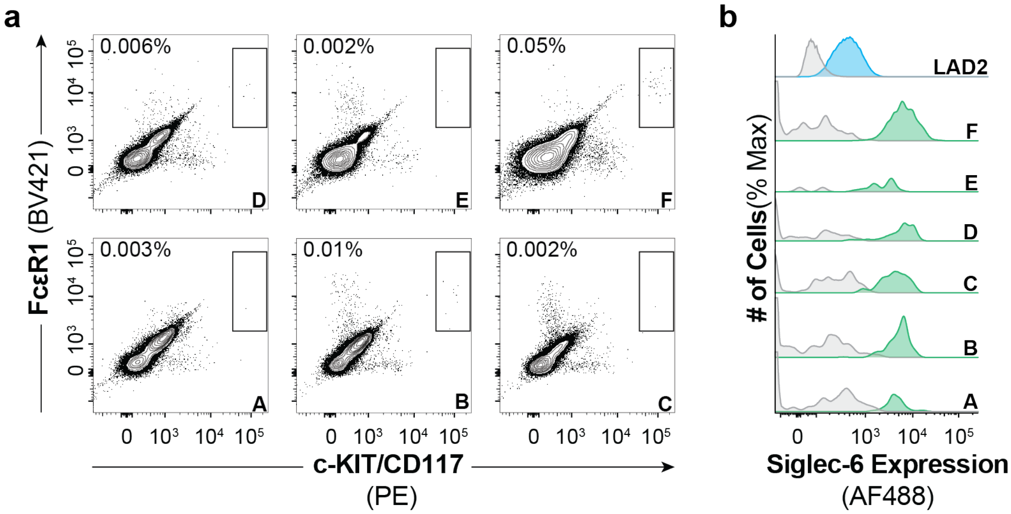

Supplementary Fig. 30: Characterization of expression of Siglec-6 of primary mast cells isolated from human spleens. a, Mast cells from six different healthy donors (represented by capital letters) identified as FcεR1 and c-KIT/CD117 positive. Percentages of mast cells in total white blood cells are in upper left corner of each plot. **b**, Representative flow cytometry of Siglec-6 expression on LAD2 cells and primary mast cells stained with anti-Siglec-6-AF488.

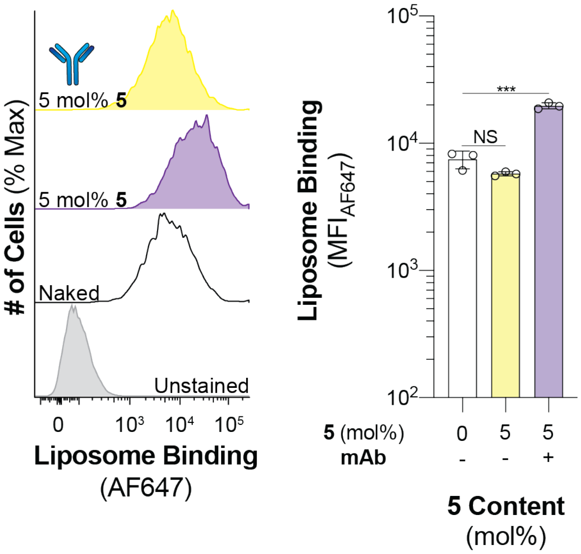

Supplementary Fig. 31: Blocking of 5 nGLL binding to LAD2 cells with anti-Siglec-6 antibody. LAD2 were pre-incubated with anti-Siglec-6 antibody prior to incubation with **5** nGLLs followed by analysis by flow cytometry. Data is presented with a representative flow cytometry histogram and was quantified as the mean ± one standard deviation of the median fluorescent intensity (MFI) from three technical replicates. A Brown-Forsythe and Welch one-way ANOVA was used to compare **5** liposome binding to naked liposome binding. Not Significant (NS); *** = 0.001 > P ≥ 0.0001.

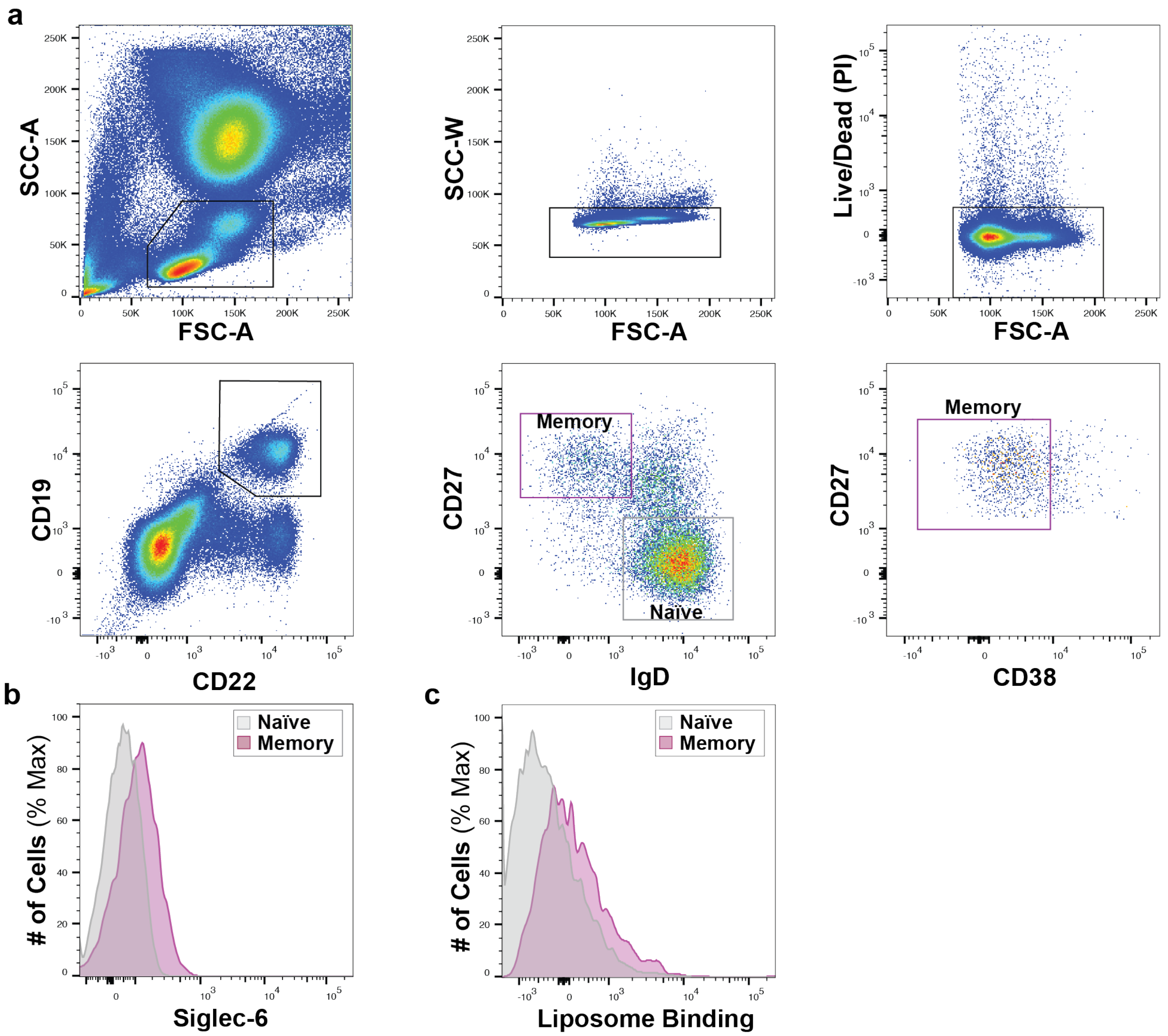

Supplementary Fig. 32: Binding of nGLLs to peripheral blood memory B-cells. a, Gating scheme for defining Memory B-cells (CD19⁺, CD22⁺, CD27⁺, IgD⁻, CD38⁻) and naïve B-cells (CD19⁺, CD22⁺, CD27⁻, IgD⁺) from peripheral blood mononuclear cells (PMBCs). **b** and **c, 5** nGLL were incubated with PBMCs and analyzed for Siglec-6 expression (**b**) and liposome binding (**c**). Representative dot plots and histograms are presented from one biological replicate.

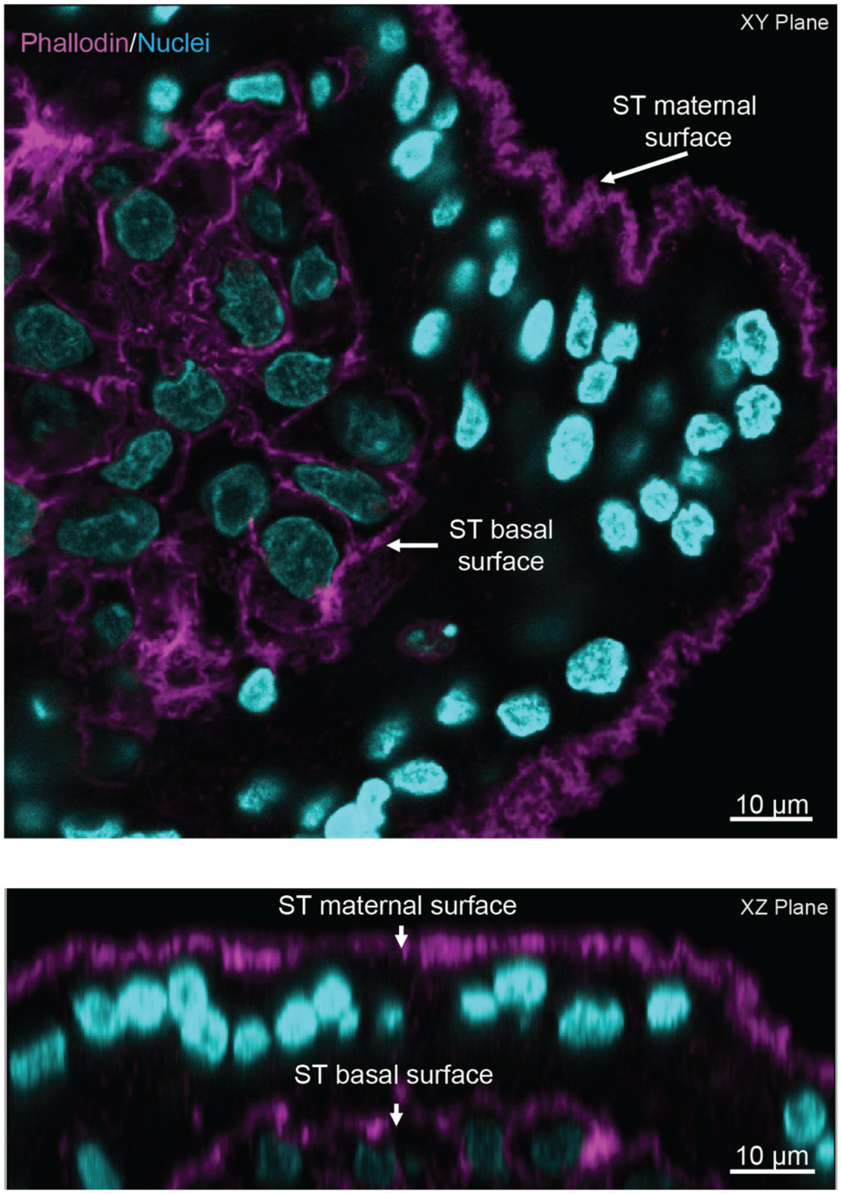

Supplementary Fig. 33: Identification of human syncytiotrophoblasts based on F-actin staining pattern in explant cultures. Representative images showing the unique F-actin (phalloidin; purple) structure of the apical surface of the first trimester human syncytiotrophoblast in cultured tissue explants (n=1 technical replicate). The syncytiotrophoblast maternal surface has a characteristic convoluted and highly branched F-actin structure that is distant from and structurally distinct from underlying mononuclear cytotrophoblast progenitors.

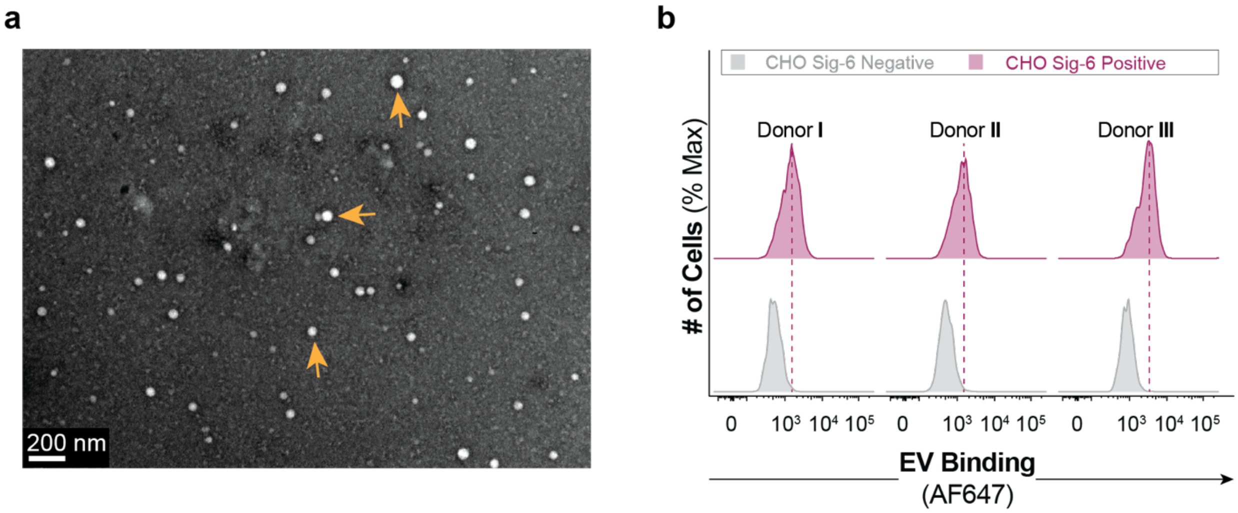

Supplementary Fig. 34: **Characterization of extracellular vesicles. a**, Transmission electron microscopy image of EVs isolated from peripheral human blood (n=1 technical replicate)**. b**, Flow cytometry histograms of isolated EVs from three different donors labeled with AF647 to CHO cells expressing WT Siglec-6.

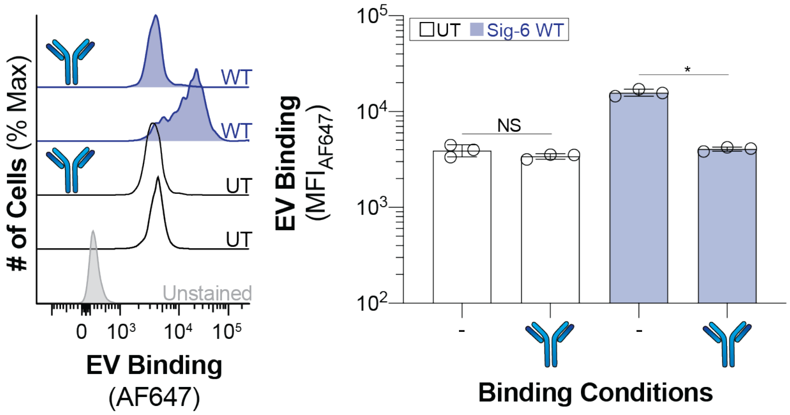

Supplementary Fig. 35: **Binding of EVs to untransfected CHO cells and CHO cells expressing WT Siglec-6 after being blocked with anti-Siglec-6.** Data is presented with a representative flow cytometry histogram and was quantified as the mean ± one standard deviation of the median fluorescent intensity (MFI) from three technical replicates. A Brown-Forsythe and Welch one-way ANOVA was used to compare **5** liposome binding before and after treatment with the anti-Siglec-6 antibody to Siglec-6 expressing CHO cells. Not Significant (NS); P > 0.5; * = 0.05 > P ≥ 0.01.

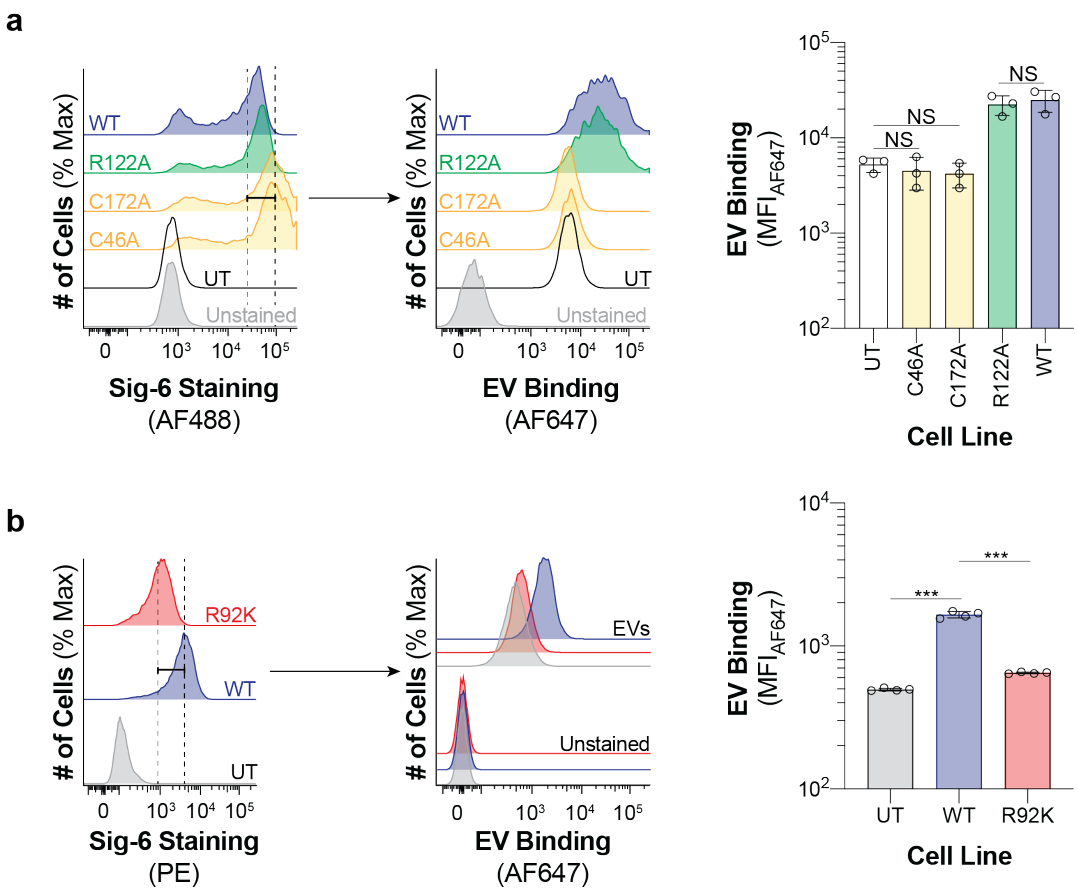

Supplementary Fig. 36: **Binding of EVs to wildtype and mutant Siglec-6 expressing CHO cells. a** and **b**, expression levels and EV binding to wildtype and C46A, R122A, C172A (n=3 technical replicates) and R92K (n=4 technical replicates) Siglec-6 expressing CHO cells respectively. Data is presented with a representative flow cytometry histogram and was quantified as the mean ± one standard deviation of the median fluorescent intensity (MFI) from at least three technical replicates. For panels **a** and **b**, a Brown-Forsythe and Welch one-way ANOVA was used for statical analysis. Not Significant (NS); *** = 0.001 > P ≥ 0.0001.

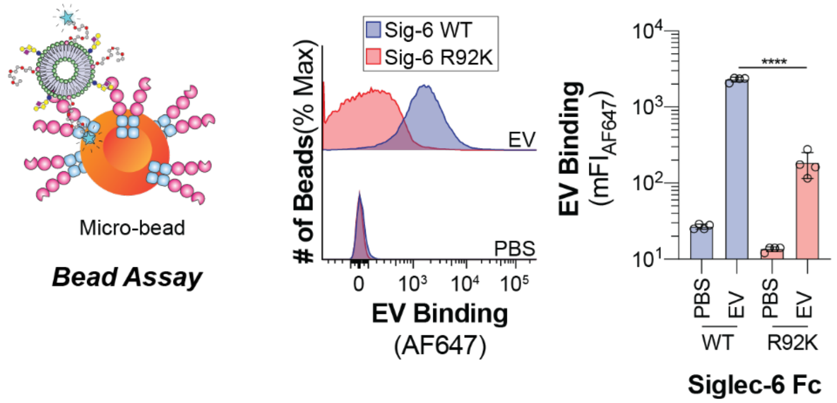

Supplementary Fig. 37: Binding of EVs to Siglec-6 R92K-Fc in bead assay. Data is presented with a representative flow cytometry histogram and was quantified as the mean ± one standard deviation of the mean fluorescent intensity (mFI) from four technical replicates. A Brown-Forsythe and Welch one-way ANOVA was used to compare the binding of 5% 5 nGLLs between WT and R92K Siglec-6. **** = $P < 0.0001$.

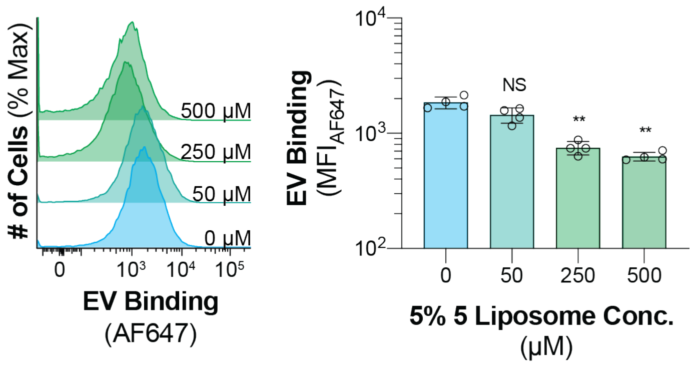

Supplementary Fig. 38: Binding competition between EVs and 5 nGLLs against WT Siglec-6. Binding of EVs to WT Siglec-6 in the presence of an increasing amount of 5 mol% **5** nGLLs. Data is presented with a representative flow cytometry histogram and the median fluorescent intensity of each replicate. The black dashed line represents the mean MFI of the naked liposomes. Data is presented with a representative flow cytometry histogram and was quantified as the mean ± one standard deviation of the media fluorescent intensity (MFI) from four technical replicates. A Brown-Forsythe and Welch one-way ANOVA was used to compare the binding of liposomes formulated with nGLLs to naked liposomes. Not Significant (NS); P > 0.5; ** = 0.01 > P ≥ 0.001.

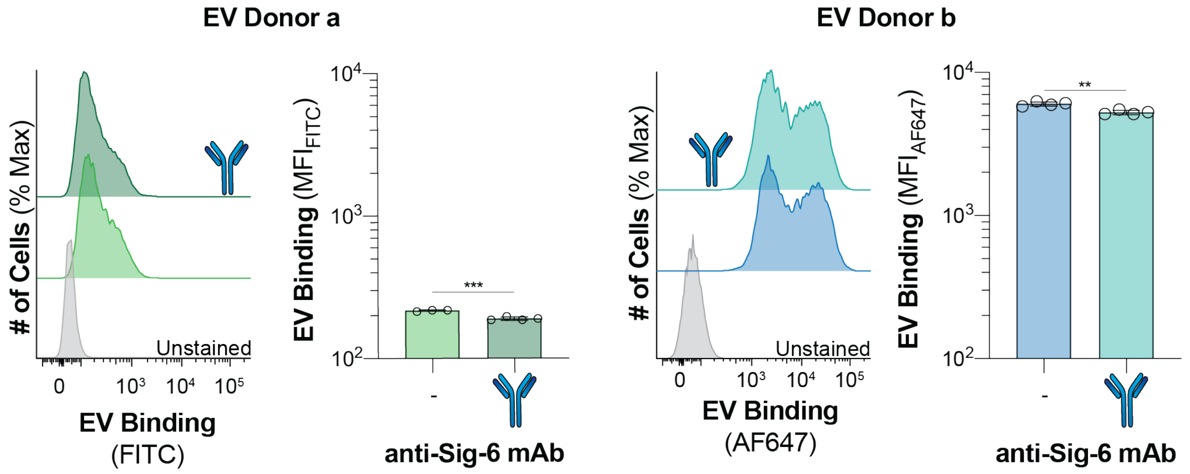

Supplementary Fig. 39: Blocking of EV binding from two different donors to LAD2 cells with anti-Siglec-6 antibody. LAD2 were pre-incubated with anti-Siglec-6 antibody prior to incubation with EVs followed by analysis by flow cytometry. Data is presented with a representative flow cytometry histogram and was quantified as the mean ± one standard deviation of the median fluorescent intensity (MFI) from four technical replicates. A two tailed Student's t-test was used for statistical analysis. ** = 0.01 > P ≥ 0.001.

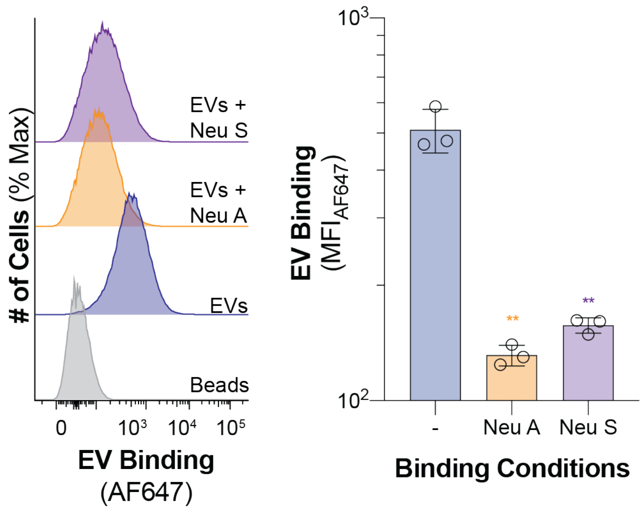

Supplementary Fig. 40: Enzymatic removal of sialic acid from EVs abrogates binding to Siglec-6. EVs were treated with neuraminidase S, neuraminidase A, or BSA prior to incubation with Streptavidin microbeads containing immobilized WT Siglec-6-Fc followed by analysis by flow cytometry. Data is presented with a representative flow cytometry histogram and was quantified as the mean ± one standard deviation of the median fluorescent intensity (MFI) from three technical replicates. A Brown-Forsythe and Welch one-way ANOVA was used to compare EV binding before and after treatment with the Neu A or Neu S to Siglec-6 expressing CHO cells. ** = $0.01 > P \geq 0.001$.

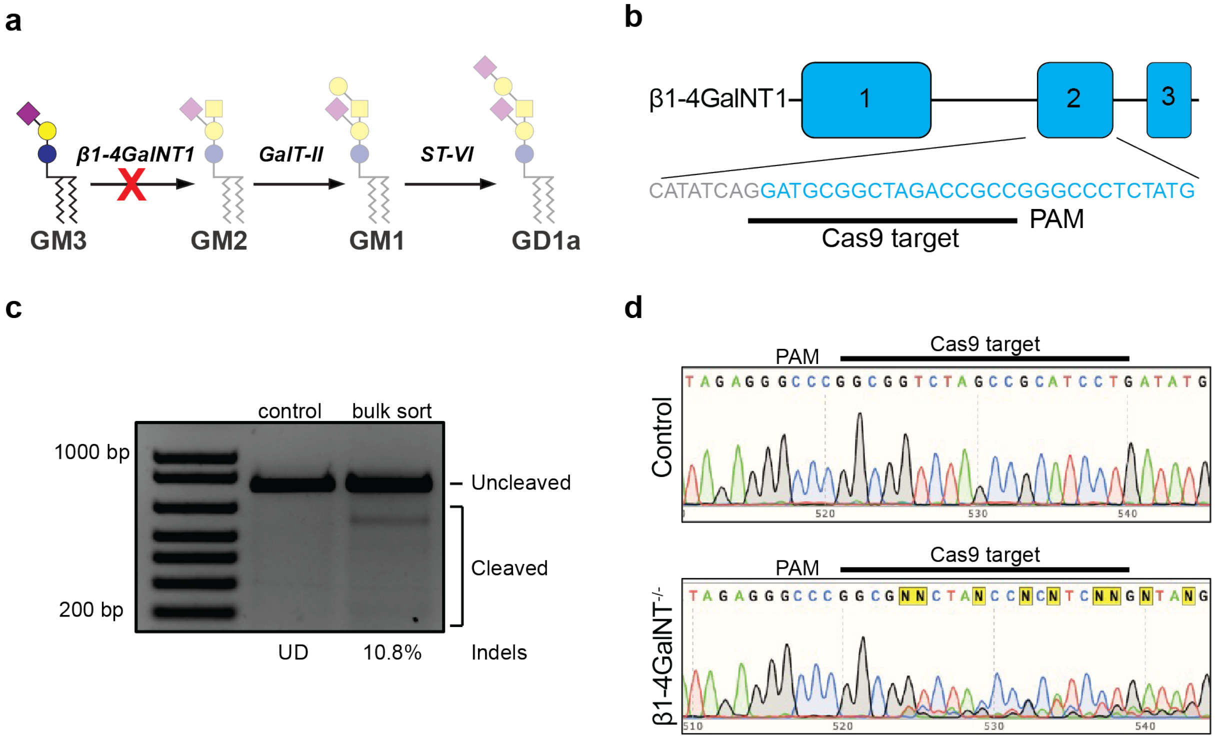

Supplementary Fig. 41: Complex gangliosides in EVs are ligands for Siglec-6. **a**, Abbreviated ganglioside biosynthesis highlighting the role of β1-4GalNT1 in ganglioside biosynthesis[12].**b**, Schematic illustration of the Cas9 target site used to generate β1-4GalNT1$^{-/-}$ cells. Intronic sequences are indicated by grey lettering, while blue lettering indicates exons. **c**, Gel showing relative cellular cleavage efficiencies of the untreated, parental cells against a population of cells FACS sorted for ATTO-550 fluorescence following Cas9 RNP transfection as determined by T7 endonuclease I digestion (n=1 technical replicate). **d**, Sanger sequencing trace of the *β1-4galnt1* target site for either parental control cells (above), or the monoclonal β1-4GALNT1$^{-/-}$ cells (below).

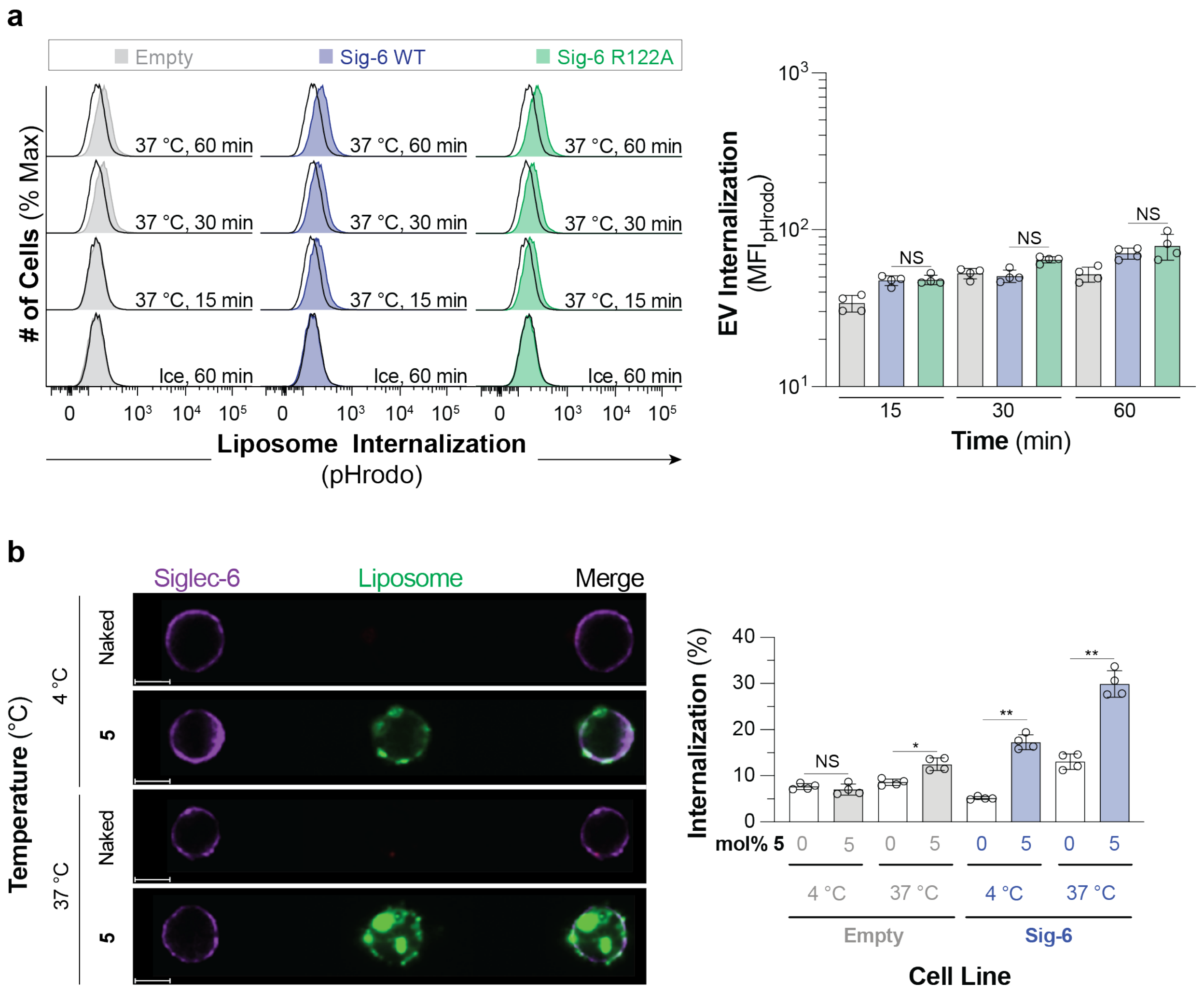

Supplementary Fig. 42: **Siglec-6 internalizes 5 GLs in Daudi cells**. **a**, Fluorescence of pHrodo labeled liposomes incubated with Daudi cells stably transduced with empty vector and WT Siglec-6 over 60 min at 4 °C or 37 °C. Data is presented with a representative flow cytometry histogram and was quantified as the mean ± one standard deviation of the median fluorescent intensity (MFI) from at least three technical replicates. **b,** Imaging flow cytometry fluorescence of Daudi cells transduced with Siglec-6 incubated with AF647-labeled liposomes (green) for 60 min at 4 °C or 37 °C. Anti-Siglec-6-AF488 antibody staining (purple) shows the cell surface expression of Siglec-6. Scale bars represent 7 μm. Imaging flow internalization data for empty vector and WT Siglec-6 virally transduced cells were quantified using IDEAS Software, version 6.2; error bars represent one standard deviation from the mean. Data is presented with a representative flow cytometry histogram and was quantified as the mean ± one standard deviation of the median fluorescent intensity (MFI) from four technical replicates. For **a** and **b**, a Brown-Forsythe and Welch one-way ANOVA was used to compare if liposomes formulated with **5** were significantly higher than a naked liposome. Not Significant (NS); P > 0.5; * = 0.05 > P ≥ 0.01; ** = 0.01 > P ≥ 0.001.

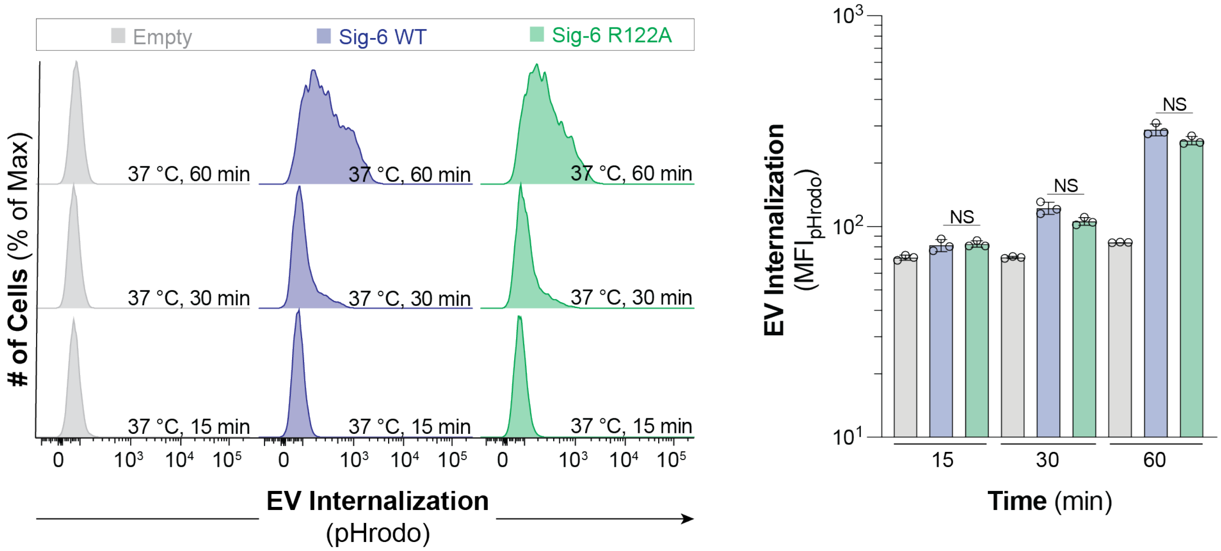

Supplementary Fig. 43: **Daudi cells internalizes EVs in a Siglec-6 dependent manner.** Daudi cell stably transduced with Siglec-6 or empty lentiviral vector were incubated with pHrodo labelled EVs for different amounts of time and analyzed by flow cytometry. Data is presented with a representative flow cytometry histogram and was quantified as the mean ± one standard deviation of the media fluorescent intensity (MFI) from three technical replicates. A Brown-Forsythe and Welch one-way ANOVA was used for statistical analysis. Not Significant (NS).

Supplementary References

1. Bhattacherjee A*, et al.* Increasing phagocytosis of microglia by targeting CD33 with liposomes displaying glycan ligands. *J Control Release* **338**, 680-693 (2021).

2. Hosoguchi K*, et al.* An efficient approach to the discovery of potent inhibitors against glycosyltransferases. *J Med Chem* **53**, 5607-5619 (2010).

3. Dziadek S, Jacques S, Bundle DR. A novel linker methodology for the synthesis of tailored conjugate vaccines composed of complex carbohydrate antigens and specific TH-cell peptide epitopes. *Chemistry* **14**, 5908-5917 (2008).

4. Nycholat CM, Rademacher C, Kawasaki N, Paulson JC. In silico-aided design of a glycan ligand of sialoadhesin for in vivo targeting of macrophages. *J Am Chem Soc* **134**, 15696-15699 (2012).

5. Han L*, et al.* Neoglycolipids as Glycosphingolipid Surrogates for Protein Binding Studies Using Nanodiscs and Native Mass Spectrometry. *Anal Chem* **92**, 14189-14196 (2020).

6. Johansson SM, Arnberg N, Elofsson M, Wadell G, Kihlberg J. Multivalent HSA conjugates of 3'-sialyllactose are potent inhibitors of adenoviral cell attachment and infection. *Chembiochem* **6**, 358-364 (2005).

7. Blixt O, Collins BE, van den Nieuwenhof IM, Crocker PR, Paulson JC. Sialoside specificity of the siglec family assessed using novel multivalent probes: identification of potent inhibitors of myelin-associated glycoprotein. *J Biol Chem* **278**, 31007-31019 (2003).

8. Yudina ON, Sherman AA, Nifantiev NE. Synthesis of propyl and 2-aminoethyl glycosides of α-d-galactosyl-(1→3′)-β-lactoside. *Carbohydrate Research* **332**, 363-371 (2001).

9. Jumper J*, et al.* Highly accurate protein structure prediction with AlphaFold. *Nature* **596**, 583-589 (2021).

10. Vasiliu D*, et al.* Large-scale chemoenzymatic synthesis of blood group and tumor-associated poly-N-acetyllactosamine antigens. *Carbohydr Res* **341**, 1447-1457 (2006).

11. Han L*, et al.* How Choice of Model Membrane Affects Protein-Glycosphingolipid Interactions: Insights from Native Mass Spectrometry. *Anal Chem* **94**, 16042-16049 (2022).

12. Yu RK, Tsai YT, Ariga T, Yanagisawa M. Structures, biosynthesis, and functions of gangliosides--an overview. *J Oleo Sci* **60**, 537-544 (2011).
