# Supplementary material for: Siglec-6 mediates the uptake of extracellular vesicles through a noncanonical glycolipid binding pocket
Source: Nat Commun. 2023 Apr 22;14:2327. doi: 10.1038/s41467-023-38030-6 (PMC10122656; doi:10.1038/s41467-023-38030-6)
Supplement: Supplementary file 3 — Reporting Summary [file 41467_2023_38030_MOESM3_ESM.pdf]

nature portfolio

Corresponding author(s): Matthew S. Macauley

Last updated by author(s): Mar 30, 2023

Reporting Summary

Nature Portfolio wishes to improve the reproducibility of the work that we publish. This form provides structure for consistency and transparency in reporting. For further information on Nature Portfolio policies, see our Editorial Policies and the Editorial Policy Checklist.

Statistics

For all statistical analyses, confirm that the following items are present in the figure legend, table legend, main text, or Methods section.

n/a	Confirmed	
☐	☒	The exact sample size (*n*) for each experimental group/condition, given as a discrete number and unit of measurement
☐	☒	A statement on whether measurements were taken from distinct samples or whether the same sample was measured repeatedly
☐	☒	The statistical test(s) used AND whether they are one- or two-sided<br>*Only common tests should be described solely by name; describe more complex techniques in the Methods section.*
☒	☐	A description of all covariates tested
☒	☐	A description of any assumptions or corrections, such as tests of normality and adjustment for multiple comparisons
☐	☒	A full description of the statistical parameters including central tendency (e.g. means) or other basic estimates (e.g. regression coefficient) AND variation (e.g. standard deviation) or associated estimates of uncertainty (e.g. confidence intervals)
☐	☒	For null hypothesis testing, the test statistic (e.g. *F*, *t*, *r*) with confidence intervals, effect sizes, degrees of freedom and *P* value noted<br>*Give P values as exact values whenever suitable.*
☐	☒	For Bayesian analysis, information on the choice of priors and Markov chain Monte Carlo settings
☒	☐	For hierarchical and complex designs, identification of the appropriate level for tests and full reporting of outcomes
☒	☐	Estimates of effect sizes (e.g. Cohen's *d*, Pearson's *r*), indicating how they were calculated

Our web collection on statistics for biologists contains articles on many of the points above.

Software and code

Policy information about availability of computer code

Data collection	Flow cytometry performed on: BD FACSDivaTM software Version 8.0.1<br>Absorbance and Relative Fluorescence Units performed on Molecular Devices Soft Max Pro 7.0.3<br>Dynamic Light Scattering was performed on Malvern Panalytical Zetasizer software<br>Xcalibur (Thermo Fisher Scientific, version 4.4)
Data analysis	Volocity software version 6.3 from Quorum technologies<br>Graphpad Prism 8.4<br>FlowJo LLC. Version 10.5.3.

For manuscripts utilizing custom algorithms or software that are central to the research but not yet described in published literature, software must be made available to editors and reviewers. We strongly encourage code deposition in a community repository (e.g. GitHub). See the Nature Portfolio guidelines for submitting code & software for further information.

Data

Policy information about availability of data

All manuscripts must include a data availability statement. This statement should provide the following information, where applicable:

- Accession codes, unique identifiers, or web links for publicly available datasets
- A description of any restrictions on data availability
- For clinical datasets or third party data, please ensure that the statement adheres to our policy

The authors declare that all data supporting the findings of this study are available within the paper and its supplementary information files. Please contact the corresponding author Dr. Matthew S. Macauley (macauley@ualberta.ca) for questions regarding the raw data. Further information will be made available upon reasonable request.

Human research participants

Policy information about studies involving human research participants and Sex and Gender in Research.

Reporting on sex and gender	We did not report on sex or gender on any of the human experiments as our ethics protocol did not allow for the collection of this information.
Population characteristics	Participants were chosen at random and were used as healthy controls. Information regarding their health, age, gender, or sex was not recorded.
Recruitment	Participants were chosen on a volunteer basis. Selection biases are not applicable as all samples were healthy controls and are not being compared to other groups.
Ethics oversight	All experiments involving human blood samples and placental sample collection were approved by the human research ethics board (HREB) biomedical panel at the University of Alberta.

Note that full information on the approval of the study protocol must also be provided in the manuscript.

Field-specific reporting

Please select the one below that is the best fit for your research. If you are not sure, read the appropriate sections before making your selection.

☒ Life sciences ☐ Behavioural & social sciences ☐ Ecological, evolutionary & environmental sciences

For a reference copy of the document with all sections, see nature.com/documents/nr-reporting-summary-flat.pdf

Life sciences study design

All studies must disclose on these points even when the disclosure is negative.

Sample size	Sample size was for each experiment was influenced by sample availability however at least three biological replicates were used in each experiment involving human samples.<br><br>-For human blood samples, four biological replicates were used to measure liposome binding.<br>-For human placental samples at least three biological replicates were used for each experiment.<br>-For human spleen experiments, six biological replicates were used.
Data exclusions	No data was excluded from this study.
Replication	At least three analytical replicates were done for each experiment.<br>At least three biological replicates each containing at least three technical replicates were performed for each human blood, placental, and spleen sample.<br>All attempts at replication were successful.
Randomization	No clinical trials were performed in this study so randomization was not necessary.
Blinding	No clinical trials were performed in this study so blinding was not necessary.

Reporting for specific materials, systems and methods

We require information from authors about some types of materials, experimental systems and methods used in many studies. Here, indicate whether each material, system or method listed is relevant to your study. If you are not sure if a list item applies to your research, read the appropriate section before selecting a response.

Materials & experimental systems

n/a	Involved in the study
☐	☒ Antibodies
☐	☒ Eukaryotic cell lines
☒	☐ Palaeontology and archaeology
☒	☐ Animals and other organisms
☒	☐ Clinical data
☒	☐ Dual use research of concern

Methods

n/a	Involved in the study
☒	☐ ChIP-seq
☐	☒ Flow cytometry
☒	☐ MRI-based neuroimaging

Antibodies

Antibodies used

For flow cytometry analysis we used: anti-human CD169 (clone 7-239, PE, Biolegend, cat. no. 346003), anti-human CD22 (clone HIB22, PE, Biolegend, cat. no. 302506), anti-human CD33 (clone WM53, PE, Biolegend, cat. no. 983904), anti MAG (clone A-11, PE, Santa Cruz, cat. no. sc-166849 PE), anti-Siglec-5 (clone 1A5, PE, Biolegend, cat. no. 352003), anti-Siglec-6 (clone 767329, PE, R&D Systems, cat. no. FAB2859P), anti-Siglec-6 (clone, 767329, unlabeled, R&D Systems, cat. no. MAB2859-SP), anti-Siglec-6 (clone, AF594, R&D Systems, cat. no. FAB2859T-100UG), anti-CD328 (clone 6-434, PE, Biolegend, cat. no. 339203), anti-Siglec-8 (clone 7C9, PE, Biolegend, cat. no. 347103), anti-Siglec-9 (clone K8, PE, Biolegend, cat. no. 351503), anti-Siglec-10 (clone 5G6, PE, Biolegend, cat. no. 347603), anti-Siglec-11 (clone 4C4, unlabeled, Biolegend, cat. no. 681702), anti-Siglec-15 (unlabeled, non-commercial), anti-mouse IgG1 (clone RMG1-1, PE, Biolegend, cat. no. 406607), anti-mouse IgG2b (clone RMG2b-1, PE, Biolegend, cat. no. 406707), Donkey anti-Mouse IgG (AF594, thermofisher, cat. no. A-21203), anti-CD19 (clone HIB19, AF488, Biolegend, cat. no. 302219), anti-CD3 (clone UCHT1, BV605, Biolegend, cat. no. 317321), anti-CD27 (clone M-T271, PerCP/Cyanine5.5, Biolegend, cat. no. 348207), anti-CD38 (clone HB-7, BV650, Biolegend, cat. no 356619), anti-CD22 (clone S-HCL-1, BV421, Biolegend, cat. no. 302523), anti-CD117 (clone S18022G, PE, Biolegend, cat. no. 332203), anti-FcεR1α (clone AER-37, BV421, Biolegend, cat. no. 334623), anti-CD34 (clone 581, BV510, Biolegend, cat. no. 343527), anti-CD19 (clone SJ25C1, APC/Cyanine7, Biolegend, cat. no. 302217), CD14 (clone M5E2, BV605, Biolegend, cat. no. 301833), anti-CD3 (clone OKT3, BV650, Biolegend, cat. no. 317323) anti-Siglec-8 (clone 7C9, PE/Cyanine7, Biolegend, cat. no. 347111a).

Validation

All of the antibodies except for anti-Siglec-15 were commercially purchased from the following companies according to the links provided : 1)https://www.biolegend.com/fr-ch, 2) https://www.scbt.com/home, 3) https://www.rndsystems.com, 4).https://www.thermofisher.com/ca/en/home.html. Commercial antibodies were quality control tested by immunofluorescence staining with flow cytometric analysis by the companies. The anti-Siglec-15 antibody was validated by staining anti Siglec-15 expressing cells. More information regarding anti-Siglec-15 can be found at https://doi.org/10.1093/glycob/cwm049.

Eukaryotic cell lines

Policy information about cell lines and Sex and Gender in Research

Cell line source(s)	Chinese Hamster Ovary, Daudi, HEK293T, LAD2, N2a
Authentication	All the parental cell lines were purchased from ATCC, cell lines were not authenticated within the lab.
Mycoplasma contamination	No Mycoplasma contamination detected (ABM Mycoplasma detection kit, Canada).
Commonly misidentified lines (See ICLAC register)	No commonly misidentified cell lines were used in the study.

Flow Cytometry

Plots

Confirm that:

☒ The axis labels state the marker and fluorochrome used (e.g. CD4-FITC).

☒ The axis scales are clearly visible. Include numbers along axes only for bottom left plot of group (a 'group' is an analysis of identical markers).

☒ All plots are contour plots with outliers or pseudocolor plots.

☒ A numerical value for number of cells or percentage (with statistics) is provided.

Methodology

Sample preparation

For adherent cell lines studied, cells were removed from the plate using 1 mM EDTA/PBS and centrifuged at 300 rcf for 5 minutes before staining. All flow samples were run in flow buffer (HBSS containing Calcium and Magnesium, 0.1% BSA) or FACS buffer (HBSS containing 1% FBS and 500 μM EDTA).

March 2021

Instrument	BD LSRFortessa TM X-20
Software	BD FACSDivaTM software V8.0.1
Cell population abundance	At least 10,000 cells were collected for staining, binding and internalization studies.
Gating strategy	Cells in all experiments are initially gated through SSC-A/FSC-A channels looking at live cells, then are further gated through SSC-W/FSC-A channels looking at singlet populations. For Liposome binding assay, Siglec expressing CHO cells were than gated on the Siglec positive population and the liposome binding of this population was reported.<br><br>For isolation of memory B-cells, memory B-cells are defined as CD19+, CD22+, CD27+, IgD–, CD38– and Naïve B-cells are defined as CD19+, CD22+, CD27-, IgD+.<br><br>For isolation of mast cells, mast cells were defined as CD117+ FcεR1+.

☒ Tick this box to confirm that a figure exemplifying the gating strategy is provided in the Supplementary Information.
